# Neuroanatomical dimensions in medication-free individuals with major depressive disorder and treatment response to SSRI antidepressant medications or placebo

**DOI:** 10.1038/s44220-023-00187-w

**Published:** 2024-01-12

**Authors:** Cynthia H. Y. Fu, Mathilde Antoniades, Guray Erus, Jose A. Garcia, Yong Fan, Danilo Arnone, Stephen R. Arnott, Taolin Chen, Ki Sueng Choi, Cherise Chin Fatt, Benicio N. Frey, Vibe G. Frokjaer, Melanie Ganz, Beata R. Godlewska, Stefanie Hassel, Keith Ho, Andrew M. McIntosh, Kun Qin, Susan Rotzinger, Matthew D. Sacchet, Jonathan Savitz, Haochang Shou, Ashish Singh, Aleks Stolicyn, Irina Strigo, Stephen C. Strother, Duygu Tosun, Teresa A. Victor, Dongtao Wei, Toby Wise, Roland Zahn, Ian M. Anderson, W. Edward Craighead, J. F. William Deakin, Boadie W. Dunlop, Rebecca Elliott, Qiyong Gong, Ian H. Gotlib, Catherine J. Harmer, Sidney H. Kennedy, Gitte M. Knudsen, Helen S. Mayberg, Martin P. Paulus, Jiang Qiu, Madhukar H. Trivedi, Heather C. Whalley, Chao-Gan Yan, Allan H. Young, Christos Davatzikos

**Affiliations:** 1 School of Psychology, University of East London, London, UK; 2 Centre for Affective Disorders, Department of Psychological Medicine, Institute of Psychiatry, Psychology, and Neuroscience, King’s College London, London, UK; 3 Center for Biomedical Image Computing and Analytics, Perelman School of Medicine, University of Pennsylvania, Philadelphia, PA USA; 4 Rotman Research Institute, Baycrest Centre, Toronto, Ontario Canada; 5 Huaxi MR Research Center, Department of Radiology, West China Hospital, Sichuan University, Chengdu, China; 6 Research Unit of Psychoradiology, Chinese Academy of Medical Sciences, Chengdu, China; 7 Nash Family Center for Advanced Circuit Therapeutics, Icahn School of Medicine at Mount Sinai, New York, NY USA; 8 Department of Psychiatry, Center for Depression Research and Clinical Care, University of Texas Southwestern Medical Center, Dallas, TX USA; 9 Department of Psychiatry and Behavioural Neurosciences, McMaster University, Hamilton, Ontario Canada; 10 Mood Disorders Treatment and Research Centre and Women’s Health Concerns Clinic, St Joseph’s Healthcare Hamilton, Hamilton, Ontario Canada; 11 Neurobiology Research Unit, University Hospital Rigshospitalet, Copenhagen, Denmark; 12 Department of Clinical Medicine, Faculty of Health and Medical Sciences, University of Copenhagen, Copenhagen, Denmark; 13 Department of Psychiatry, Psychiatric Centre Copenhagen, Copenhagen, Denmark; 14 Department of Computer Science, University of Copenhagen, Copenhagen, Denmark; 15 Department of Psychiatry, University of Oxford, Oxford, UK; 16 Oxford Health NHS Foundation Trust, Warneford Hospital, Oxford, UK; 17 Mathison Centre for Mental Health Research and Education, University of Calgary, Calgary, Alberta Canada; 18 Department of Psychiatry, Cumming School of Medicine, University of Calgary, Calgary, Alberta Canada; 19 Department of Psychiatry, University Health Network, Toronto, Ontario Canada; 20 Division of Psychiatry, Royal Edinburgh Hospital, University of Edinburgh, Edinburgh, UK; 21 Department of Radiology, Taihe Hospital, Hubei University of Medicine, Shiyan, China; 22 Centre for Depression and Suicide Studies, Unity Health Toronto, Toronto, Ontario Canada; 23 Meditation Research Program, Department of Psychiatry, Massachusetts General Hospital, Harvard Medical School, Boston, MA USA; 24 Laureate Institute for Brain Research, Tulsa, OK USA; 25 Penn Statistics in Imaging and Visualization Endeavor (PennSIVE) Center, Department of Biostatistics, Epidemiology and Informatics, University of Pennsylvania, Philadelphia, PA USA; 26 Department of Psychiatry, University of California San Francisco, San Francisco, USA; 27 Department of Medical Biophysics, University of Toronto, Toronto, Ontario Canada; 28 Department of Radiology and Biomedical Imaging, University of California San Francisco, San Francisco, CA USA; 29 School of Psychology, Southwest University, Chongqing, China; 30 Department of Neuroimaging, Institute of Psychiatry, Psychology and Neuroscience, King’s College London, London, United Kingdom; 31 Division of Neuroscience and Experimental Psychology, University of Manchester, Manchester, UK; 32 Department of Psychiatry and Behavioral Sciences, Emory University School of Medicine, Atlanta, GA USA; 33 Department of Psychology, Emory University, Atlanta, GA USA; 34 Department of Psychology, Stanford University, Stanford, CA USA; 35 Key Laboratory of Behavioral Science, Institute of Psychology, Chinese Academy of Sciences, Beijing, China; 36 South London and Maudsley NHS Foundation Trust, Bethlem Royal Hospital, London, UK

**Keywords:** Predictive markers, Depression

## Abstract

Major depressive disorder (MDD) is a heterogeneous clinical syndrome with widespread subtle neuroanatomical correlates. Our objective was to identify the neuroanatomical dimensions that characterize MDD and predict treatment response to selective serotonin reuptake inhibitor (SSRI) antidepressants or placebo. In the COORDINATE-MDD consortium, raw MRI data were shared from international samples (*N* = 1,384) of medication-free individuals with first-episode and recurrent MDD (*N* = 685) in a current depressive episode of at least moderate severity, but not treatment-resistant depression, as well as healthy controls (*N* = 699). Prospective longitudinal data on treatment response were available for a subset of MDD individuals (*N* = 359). Treatments were either SSRI antidepressant medication (escitalopram, citalopram, sertraline) or placebo. Multi-center MRI data were harmonized, and HYDRA, a semi-supervised machine-learning clustering algorithm, was utilized to identify patterns in regional brain volumes that are associated with disease. MDD was optimally characterized by two neuroanatomical dimensions that exhibited distinct treatment responses to placebo and SSRI antidepressant medications. Dimension 1 was characterized by preserved gray and white matter (*N* = 290 MDD), whereas Dimension 2 was characterized by widespread subtle reductions in gray and white matter (*N* = 395 MDD) relative to healthy controls. Although there were no significant differences in age of onset, years of illness, number of episodes, or duration of current episode between dimensions, there was a significant interaction effect between dimensions and treatment response. Dimension 1 showed a significant improvement in depressive symptoms following treatment with SSRI medication (51.1%) but limited changes following placebo (28.6%). By contrast, Dimension 2 showed comparable improvements to either SSRI (46.9%) or placebo (42.2%) (*β* = –18.3, 95% CI (–34.3 to –2.3), *P* = 0.03). Findings from this case-control study indicate that neuroimaging-based markers can help identify the disease-based dimensions that constitute MDD and predict treatment response.

## Abstract (web summary)

Using a large multi-center neuroimaging dataset, Fu, Antoniades et al. reveal that major depressive disorder is characterized by two neuroanatomical dimensions exhibiting distinct treatment response to placebo and selective serotonin reuptake inhibitor medications.

## Main

Major depressive disorder (MDD) is both highly prevalent and debilitating. MDD affects over 320 million people worldwide, is the main precursor of suicide, and is the leading cause of disability globally, with profound impacts on daily life, work, and relationships^1–4^. The remission rate is about 30% for the initial treatment, but 30–40% of patients continue to have significant symptoms despite full treatment trials of antidepressant medication or psychotherapy^5,6^. Individuals with MDD show significant heterogeneity in their symptoms and treatment outcomes and in the longitudinal course of the illness. We do not have any biomarkers to aid in identifying the disorder or to predict treatment response. Consequently, MDD is currently best conceptualized as a syndrome rather than a disease with a distinct pathophysiology.

Data-driven approaches can delineate the heterogeneity that constitutes the clinical diagnosis by identifying potential neurobiological dimensions. It is likely that distinct brain mechanisms underlie heterogeneous clinical presentations, treatment outcomes, and longitudinal course^7–9^. Neuroimaging subtypes might be able to quantify heterogeneity in clinical presentation and identify optimal treatment strategies best suited to distinct subtypes, including identifying treatment resistance early in the course of the illness^10^.

On the basis of functional connectivity measures, two to four MDD subtypes have been reported^11–15^. A common pattern of altered connectivity that included ventromedial prefrontal, orbitofrontal, and posterior cingulate cortices, insula, and subcortical regions was observed along with distinct patterns of functional connectivity and clinical symptom profiles in four subtypes^12^. By addressing heterogeneity, these studies reveal the potential to identify neuroimaging subtypes that constitute major depression. However, the variety of functional connectivity measures and clinical heterogeneity, namely, disparate depressive states, medication status, comorbid disorders, and forms of depression, including treatment-resistant depression, have limited interpretation and rendered the subtypes less comparable across studies^16,17^.

The high reliability of structural MRI and its derived measures could offer a marker of disease^18,19^. Initial studies were limited by small samples from single sites^20–22^. Recent multisite cohorts show classification accuracies ranging from 52% to 75%^23–25^. However, the classification outcomes have been binary (MDD versus control). The highest classification accuracy was achieved in a cohort with a formal MDD diagnosis in a current depressive episode, but the sample size was limited (230 MDD, 77 controls)^25^. In the Enhancing Neuroimaging Genetics through Meta-analysis (ENIGMA) consortium, consisting of 2,288 MDD and 3,077 controls, the classification accuracy was found to be up to 62%^23^. However, there was significant clinical heterogeneity, including treatment-resistant depression and a mixture of depressive states, symptom severities, and comorbid psychotic symptoms, and classifications require replications in independent patient cohorts. In a large but more clinically homogeneous sample, Wen et al^9^. identified two distinct dimensions in late-life depression (501 late-life depression, 495 controls), one with relatively preserved gray matter and a second with widespread atrophy and white-matter disruptions that showed an accelerated progression to Alzheimer’s disease. As predictors of treatment response, reduced baseline pre-treatment gray-matter volumes, in particular in the hippocampus and lingual gyrus, have been predictors of poorer treatment response, while increased volumes, including in the anterior and posterior cingulate cortices and middle frontal gyrus, have predicted treatment remission^20,26,27^.

In the present study, we sought to delineate heterogeneity in MDD in a large multisite consortium of raw individual magnetic resonance imaging (MRI) data with deep phenotypic characterization (COORDINATE-MDD^28^). We used a semi-supervised machine-learning method, heterogeneity though discriminative analysis (HYDRA)^29^, which defines dimensions of the disease (here MDD) using healthy controls as a reference group, thus avoiding clustering based on disease-irrelevant features. The present sample consists of raw individual structural MRI in individuals with MDD, defined by structured clinical diagnostic criteria, obtained during a current depressive episode of at least moderate severity, in first-episode or recurrent MDD, not treatment-resistant depression, and medication free (685 MDD, 699 controls). Because the consortium studies shared anonymized raw data, we are able to optimize characterization of the precise location and magnitude of effects in each participant.

Our aim was to identify whether MDD is characterized by distinct neuroanatomical patterns and to examine the relation between dimensions and treatment response. We hypothesized that the optimal solution in our sample would be two dimensions, as observed in late-life depression using structural MRI data^9^. Because we had longitudinal treatment outcomes in a subsample (359 MDD), we further examined whether the dimensions would demonstrate distinct predictive profiles for response to placebo or to selective serotonin reuptake inhibitor (SSRI) medications based on individual treatment responses. Due to the data-driven nature of the methods used here, it is difficult to predict the neuroanatomical characteristics of the subtypes that will emerge and therefore to derive a hypothesis regarding treatment outcomes in the subtypes. However, on the basis of previous findings, we hypothesized that a subtype with smaller volumes would predict a poorer response to antidepressant treatment.

## Results

### HYDRA reveals two-dimension optimal model

**Fig. 1:**
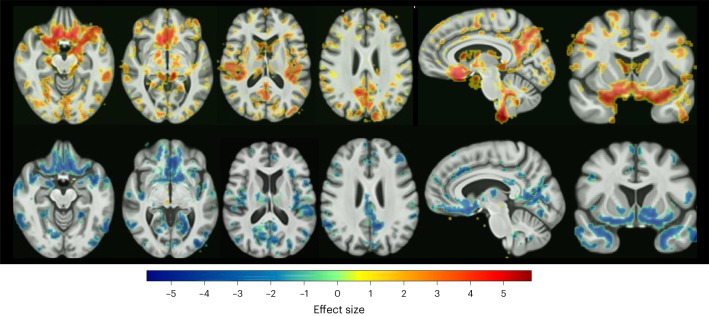
Neuroanatomical patterns across the dimensions. False discovery rate- (FDR-) corrected voxel-wise comparison of gray-matter volume differences in Dimension 1 (top row) and Dimension 2 (bottom row) versus controls are presented in transverse, sagittal, and coronal sections. Color bar indicates strength of group differences (MIDAS statistic) between MDD and healthy control participants.

The highest Adjusted Rand Index (ARI) (0.61) was achieved with a HYDRA model for *k* = 2 dimensions, consisting of 290 participants with MDD assigned to Dimension 1 (D1) and 395 participants with MDD assigned to Dimension 2 (D2). Split-sample and leave-site-out (LSO) analyses replicated the optimal *k* = 2 dimension solution. In leave-site-out analysis, the percentage overlap for MDD participants assigned to the same dimension ranged from 86.26% to 94.86% with an average overlap of 92.70%. D1 was characterized by preserved gray- and white-matter volumes in all regions relative to healthy controls, while D2 was characterized by subtle widespread decreased volumes relative to controls (Fig. 1 and Supplementary Figs. 1 and 2).

When the analysis was restricted to MDD participants in the prospective treatment trials (*N* = 359 MDD), D1 was characterized by preserved gray- and white-matter volumes, while D2 was characterized by widespread gray- and white-matter reductions compared with healthy controls, although there were no differences in anterior cingulate or hippocampal volumes.

### Clinical variables across dimensions

There were no significant differences between D1 and D2 in age of onset (*P* = 0.3), years of illness (*P* = 0.2), number of episodes (*P* = 0.07), duration of current episode (*P* = 0.9), age (*P* = 1.0), sex (*P* = 0.5), or years of education (*P* = 0.4) (Table 1).

**Table 1:** Demographic and clinical variables for MDD and healthy control participants

	Healthy controls	MDD participants	MDD D1	MDD D2
**Sample size**	699	685	290	395
**Age (yr)**	38.4 (15.4)	35.3 (12.3)	35.3 (12.6)	35.3 (12.2)
**Age range (yr)**	16–72	18–65	18–65	18–64
**Sex**				
**Female (number, percentage)**	404 (58)	439 (64)	181 (62)	258 (65)
**Male (number, percentage)**	295 (42)	246 (36)	109 (38)	137 (35)
**Ethnicity (number, percentage)**	621 (89)	470 (68)	179 (62)	290 (73)
**Asian**	167 (27)	173 (37)	80 (45)	93 (32)
**Black**	16 (3)	16 (3)	2 (1)	14 (5)
**Hispanic**	9 (1)	9 (2)	1 (0.6)	8 (3)
**Middle Eastern**	0	2 (0.4)	1 (1)	1 (0.3)
**Mixed**	10 (1.6)	10 (2)	2 (0.6)	8 (3)
**Native American**	3 (0.4)	11 (2)	1 (0.6)	10 (3)
**Pacific Islander**	1 (0.2)	2 (0.4)	1 (0.6)	1 (0.3)
**White**	415 (67)	247 (53)	91 (5)	155 (53)
**Years of education**	15.3 (2.7)	14.5 (2.7)	14.8 (2.5)	14.4 (2.8)
**HAM-D**	0.9 (1.5)	21.4 (5.1)	21.0 (4.8)	21.7 (5.3)
**MADRS**	0.5 (1.1)	29.0 (5.1)	28.9 (5.2)	29.0 (5.0)
**First-episode/recurrent MDD**		128/355	52/168	76/187
**Age of onset (yr)**		24.5 (10.8)	22.5 (9.8)	25.3 (11.1)
**Years of illness**		6.5 (9.9)	6.3 (8.8)	6.7 (10.4)
**MDD episodes**		7.7 (20.3)	8.1 (20.8)	7.4 (20.1)
**Duration of current episode (weeks)**		57.9 (119.2)	55.6 (97.7)	59.5 (132.2)
**Prospective treatment sample**				
**Total (number MDD participants)**		359	165	218
**Escitalopram**		116	38	102
**Citalopram**		36	16	20
**Sertraline**		98	56	42
**Placebo**		109	55	54
**HAM-D score**				
**Baseline**		20.5 (4.1)	20.0 (4.0)	20.9 (4.1)

Values presented are mean (s.d.) except where indicated. Montgomery–Asberg Depressive Ratings Scale (MADRS) ratings were available from CAN-BIND and Manchester samples. There is a significantly greater number of women than men participants (*P* = 0.02). Healthy controls had a higher mean age (*P* = 0.003) and greater number of years of education (*P* = 5.7 × 10^–9^) than MDD participants. Chi-squared test of independence was used for the categorical variable (sex), and the Mann–Whitney U test was used for continuous variables (age and years of education). For some sites, the number of years of education was estimated from text data; this is detailed in the Supplementary Information. One healthy control participant was 16 years old, and three were 17 years old. Treatment with SSRI antidepressants showed a significantly greater reduction in HAM-D score (post-treatment HAM-D 10.6) relative to placebo (post-treatment HAM-D 12.5) (*t* = 2.23, *P* = 0.03). Ethnicities for Stratifying Resilience and Depression Longitudinally, Oxford, Manchester Remedi, and King’s College London studies (Methods) were estimated to be around 90%, 90%, 90%, and 95% white, respectively.

### Interaction between HYDRA dimensions and treatment outcomes

Treatment with SSRI medications was associated with a significantly greater improvement in depressive symptoms (–48.7%) relative to placebo (–35.4%) across both D1 and D2 (*β* = 37.8, 95% confidence interval (CI) (12.4 to 63.1), *P* = 0.004). Treatment with SSRI antidepressants showed a significantly greater reduction in total Hamilton Depression Rating Scale (HAM-D) score (post-treatment HAM-D 10.6) relative to placebo (post-treatment HAM-D 12.5) (*t* = 2.23, *P* = 0.03).

**Fig. 2:**
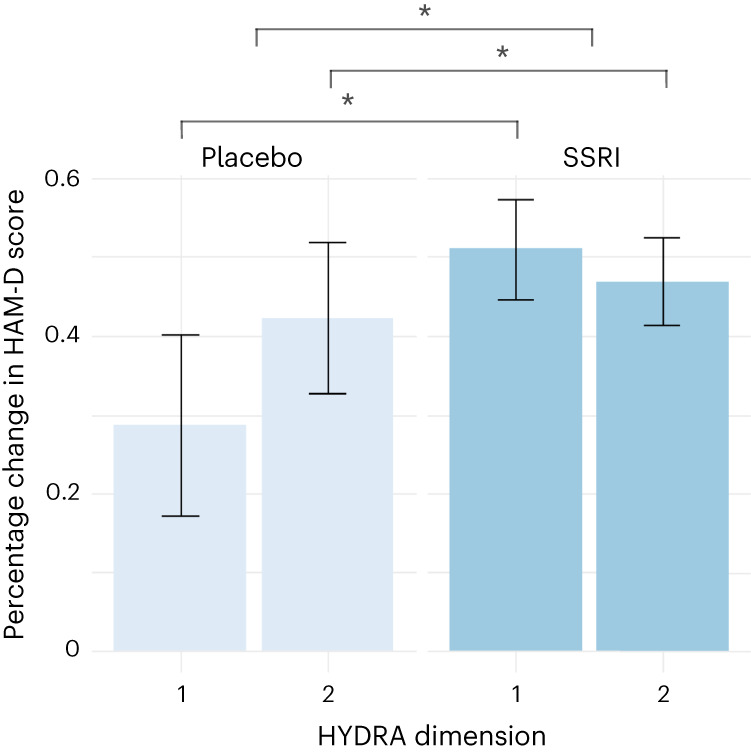
Depressive symptoms across the dimensions and treatment groups. Difference in percentage change in HAM-D scores across HYDRA dimensions (D1 (*n* = 164) and D2 (*n* = 195), *n* = 359) and binary treatment groups following treatment with SSRI medications (*n* = 250) and placebo (*n* = 109). Data are presented using a bar plot as mean values and 95th percentile error bars. The asterisks (*) indicate significant differences between the two subgroups using linear regression model (two-sided *P* < 0.05).

There was a significant dimension-by-treatment interaction effect in which D1 showed a greater improvement in depressive severity following SSRI medication (51.1%) compared with placebo (28.6%). By contrast, D2 showed a general improvement in depressive symptoms that did not achieve treatment response to either SSRI medication (46.9%) or placebo (42.2%) (*β* = –18.3, 95% CI (–34.3 to –2.3), *P* = 0.03) (Fig. 2).

To examine whether the interaction between dimensions and treatment group differed according to SSRI medication, we performed a second linear regression with the treatment group variable including all four treatment categories (SSRI sertraline, SSRI escitalopram, SSRI citalopram, and placebo) instead of a binary category (SSRI medications and placebo). The effect size (Cohen’s *f*^2^ = 0.13) of the interaction term has an *F* statistic of 4.361 based on our analysis using a linear regression model. With a sample size of 359, assuming that we adjust for 10 additional covariates in the model and the same effect size, we have over 99% power to detect a significant interaction term between treatment and HYDRA dimension under 5% Type I error. The outcome variable and covariates of the linear model remained unchanged. Treatment with citalopram (*N* = 36 MDD) was associated with the greatest improvement in symptoms compared with placebo (*N* = 109 MDD) (mean reduction = 68.8%, *β* = 74.1, 95% CI (30.0 to 118.4), *P* = 0.001), followed by escitalopram (*N* = 116 MDD) (mean reduction = 48.8%, *β* = 48.6, 95% CI (14.0 to 83.3), *P* = 0.006) and then sertraline (*N* = 98 MDD) (mean reduction = 41.3%, *β* = 41.8, 95% CI (12.8 to 70.9), *P* = 0.005).

**Fig. 3:**
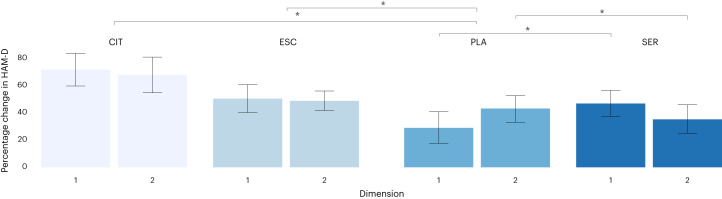
Depressive symptoms across the dimensions and all four treatment groups. Difference in percentage change in HAM-D scores across HYDRA dimensions (D1 (*n* = 164) and D2 (*n* = 195), *n* = 359) and four different treatment groups following treatment with SSRI sertraline (SER, *n* = 98), SSRI escitalopram (ESC, *n* = 116), SSRI citalopram (CIT, *n* = 36), and placebo (PLA, *n* = 109). Data are presented using a bar plot as mean values and 95th percentile error bars. The asterisks (*) indicate significant differences between the two subgroups using linear regression model (two-sided *P* < 0.05).

There was a significant interaction between dimensions and treatment response to sertraline: D1 showed a greater improvement in depression severity following sertraline treatment relative to placebo, whereas D2 showed a greater improvement in depression severity following placebo relative to sertraline (*β* = –24.6, 95% CI (–43.4 to –5.7), *P* = 0.01). There were no significant interactions between dimensions and escitalopram (*P* = 0.17) or citalopram (*P* = 0.17) (Fig. 3).

**Fig. 4:**
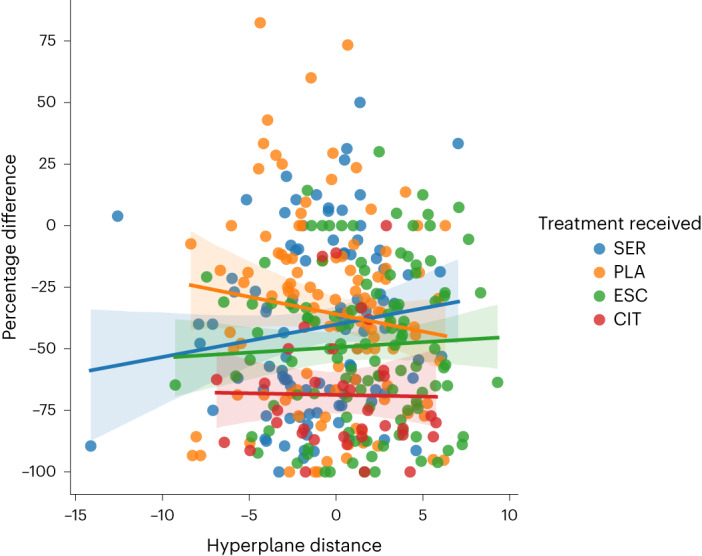
Relationship between dimension membership and change in depressive symptoms following treatment. Relationship between the support vector machine hyperplane (*x* axis) distance for each participant and the percentage change in HAM-D scores (*y* axis) following treatment with sertraline, escitalopram, citalopram, or placebo. Positive and negative values represent the distance from the hyperplane separating patients into D1 and D2. The larger the value, the more certain the classification within that dimension. Linear regression model shows a significant interaction between hyperplane distance and sertraline treatment (*β* = 2.73, *P* = 0.046 (two-sided), 95% CI (0.04 to 5.4). The shaded areas represent the 95% confidence intervals.

In the machine-learning analysis with linear regression using the calculated hyperplane distance in place of the binary dimension label, we similarly found that treatment response to placebo tended to increase with likelihood of being clustered in D2, while response to sertraline tended to decrease (Fig. 4).

### Case-control comparisons of gray-matter volume

**Fig. 5:**
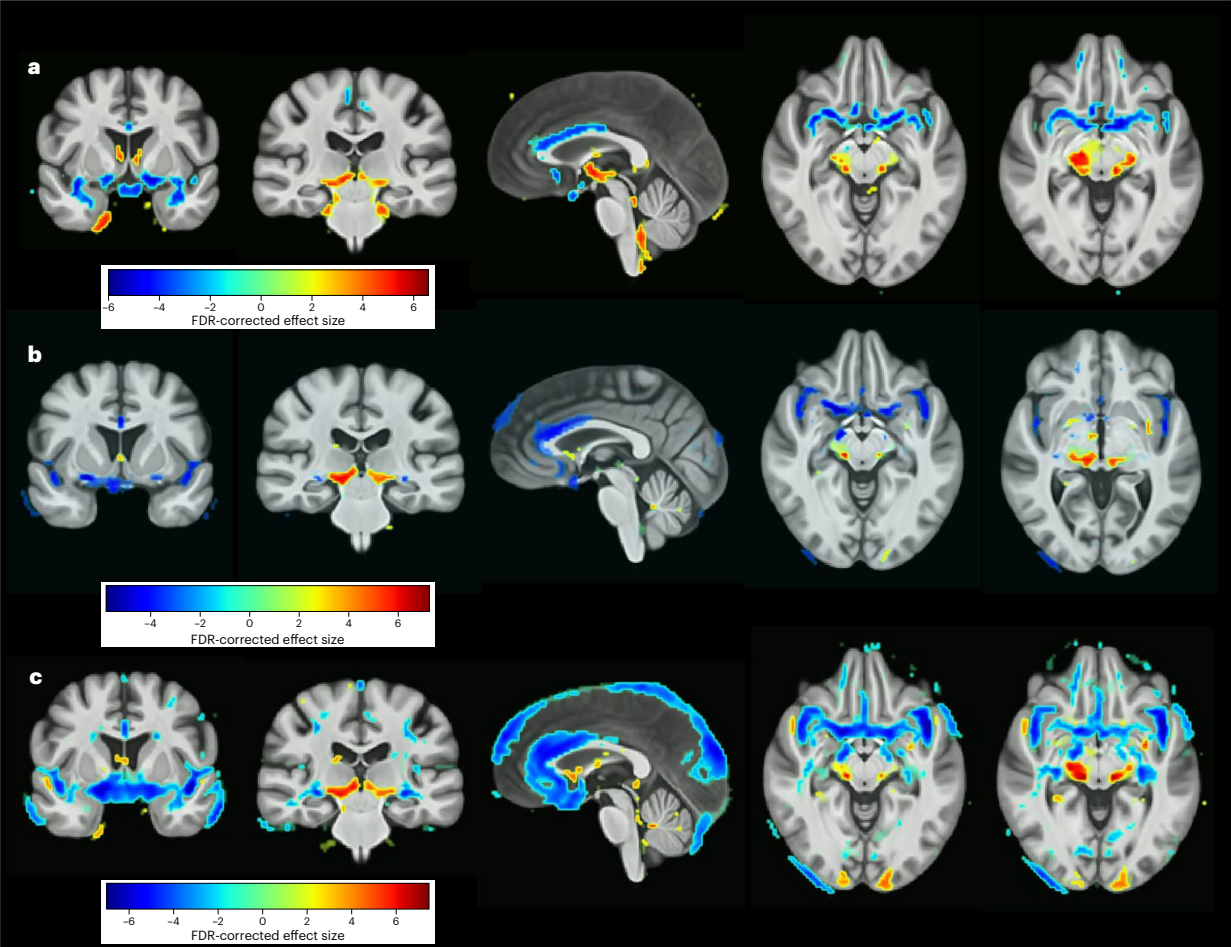
Neuroanatomical case-control differences. **a**,**b**, FDR-corrected voxel-wise comparison of gray-matter volume differences between the whole MDD participant group versus healthy controls (**a**) and after controlling for medication status (**b**). **c**, Gray-matter volume differences between MDD participants in a first episode of depression and healthy controls. The color bars indicate the strength of the group differences (MIDAS statistic) between MDD and healthy control participants.

The voxel-wise regional analysis of volumes in normalized space (RAVENS) showed several areas of significant gray-matter volume reductions in MDD participants relative to healthy controls, including in bilateral medial orbital gyri, bilateral subgenual, pregenual and dorsal anterior cingulate cortices, and bilateral insula. Significant gray-matter volume increases were evident in MDD participants relative to healthy controls in the left parahippocampal gyrus, bilateral ventral diencephalon, and extended into the left brainstem (Fig. 5a).

Controlling for medication history or recurrent MDD as a proxy measure of previous medication use, significant gray-matter volume reductions remained in the anterior cingulate and insula, and additional gray-matter volume reductions became significant, including in the right superior frontal gyrus, left parahippocampal gyrus, bilateral basal forebrain, and left cuneus (Fig. 5b). No regions showed significantly increased volumes in MDD relative to healthy controls. Furthermore, after excluding MDD participants with recurrent depression, MDD participants in a first episode of depression (*n* = 262) showed more-pronounced gray-matter reductions in the same regions, in particular in the bilateral anterior cingulate, frontal pole, medial frontal gyri, middle frontal gyri, gyrus rectus, orbital gyri, insula, inferior and superior temporal gyri, as well as bilateral lingual gyri (Fig. 5c).

## Discussion

In the present study, MDD was characterized by two reproducible neuroanatomical dimensions that showed distinct responses to placebo and SSRI antidepressant medications. D1 demonstrated preserved regional volumes compared with healthy controls and significantly greater treatment responses to SSRI antidepressants relative to placebo. By contrast, D2 was characterized by widespread volumetric reductions and no significant differences in the clinical response to placebo or SSRI antidepressants. The dimensions were revealed using a fully data-driven analysis in a large multisite consortium consisting of raw individual data from deeply phenotyped MDD individuals who were medication free with first-episode or recurrent MDD, not treatment-resistant depression, and who were in a current depressive episode of at least moderate severity without psychotic features.

Early classification studies were hampered by small sample sizes from a single site^20,21^. While recent studies have included large multisite sample sizes, only binary case-control classification has been achieved using structural MRI, perhaps limited by clinical heterogeneity in the MDD samples^23,24^. In a more clinically homogeneous MDD sample that was in a current depressive episode, a higher accuracy was achieved, but this was also a binary case-control classification, which could be due to the limited sample size^25^. The present study sought to address these two issues of size and clinical heterogeneity in a large multisite sample and relatively homogeneous deeply phenotyped clinical cohorts, which revealed two neuroanatomical dimensions.

Dimension D1 showed generally preserved neuroanatomy, while D2 showed widespread decreased volumes. In D2, the greatest deficits were observed in the insula, limbic, and temporal lobes. Volumetric predictors of clinical response in major depression have included the left middle frontal and right angular gyri for treatment with SSRI medications, escitalopram or sertraline, or to the serotonin and noradrenaline reuptake inhibitor (SNRI), venlafaxine, in the International Study to Predict Optimized Treatment in Depression study^30^, increased hippocampal tail volumes for the SSRI medication, escitalopram, in the Canadian Biomarker Integration Network in Depression (CAN-BIND) study^31^, as well as anterior and posterior cingulate cortex and left middle frontal gyri for the SSRI medication, fluoxetine^20^. The present findings indicate that widespread preserved neuroanatomy in MDD might further distinguish clinical response to either SSRI medications or to placebo. Furthermore, early changes observed after a week of treatment (for example, increased anterior cingulate cortical thickness being associated with better clinical responses to the SSRI, sertraline, in the Establishing Moderators and Biosignatures of Antidepressant Response in Clinical Care (EMBARC) study^32^ and increased hippocampal volume being associated with improved clinical responses to the SNRI, duloxetine^33^) could provide additional predictive markers and suggest potential mechanisms.

The whole-brain case-control analysis of gray-matter volumes revealed reductions in the anterior cingulate, medial orbital gyri, and insula. In first-episode MDD, gray-matter reductions were observed more widely in bilateral anterior cingulate, medial and middle frontal gyri, gyrus rectus, orbital gyri, insula, and inferior and superior temporal gyri. Meta-analyses have reported widespread gray-matter deficits from the anterior cingulate, medial prefrontal and orbitofrontal cortices, insula, hippocampus, parietal, and temporal regions in recurrent MDD^34^ with more-limited reductions in first-episode MDD, including the anterior cingulate, gyrus rectus, medial orbital gyri, and temporal gyri^35^. In the ENIGMA-MDD consortium, widespread reductions were found in cortical gray matter, which included the orbitofrontal cortex, anterior and posterior cingulate, insula, and temporal lobes^36^. Recent meta-analyses have also reported regional increases in cortical thickness in the anterior cingulate, posterior cingulate, ventromedial prefrontal, and orbitofrontal and supramarginal cortices^37,38^, which are evident in medication-free MDD^37^ and predominantly in first-episode medication-naïve MDD^37–40^. While cortical gray matter is the product of cortical thickness and surface area, which have distinct genetic and developmental origins^41^, gray-matter volume is more affected by surface area^42^. The regional distributions include the medial prefrontal–limbic network, which is posited to be important for affective regulation and modulated by serotonin function^43^ as well as the orbitofrontal–striatal network implicated in reward processing and modulated by dopamine function^44^.

The mechanisms for increased volumes could reflect disease-related as well as compensatory responses. Synaptic pruning is a fundamental process in brain development and maturation^45^. Neuron–glial cell signaling has a crucial role in synaptic pruning, which can strengthen more active synapses and remove less-active connections, improving neuronal signal-to-noise ratio^45^, while aberrant pruning might contribute to neurodevelopmental disorders. Compensatory responses include structural plasticity as an adaptive response to a neural insult, resulting in increases in activity, such as hyperexcitability in connected areas with increased synaptogenesis that can be observed in morphometric changes^46^.

Altered immune activation and inflammatory responses have been documented in MDD, including hypothalamic–pituitary–adrenal- (HPA-) axis hyperactivity. Prefrontal gray-matter volumes have shown an inverse relation with serum levels of high-sensitivity C-reactive protein^47^, and an inverse correlation has been found for orbitofrontal cortical thickness with interleukin-6^48^ as well as serum cortisol in MDD^49^. Inflammatory responses, neurotransmitter levels, and neurotrophic factors further modify neuronal and glial cells, which might be more subtle for neuronal cell bodies relative to glial cell density^50^. Elevated levels of inflammation, however, are most evident in treatment-resistant depression^51^, while the present sample consisted of first-episode and recurrent MDD.

Functional connectivity within intrinsic brain networks offers complementary measures. Reduced baseline resting-state connectivity within the orbitofrontal component of the default mode network (DMN) has been found to predict clinical response to the antidepressant medication duloxetine^33^. Pre-treatment connectomic signatures within the DMN as well as inter-network connectivity distinguished MDD participants who achieve remission with antidepressant medication and those with persistent symptoms^52^. There were no significant differences between the antidepressant medication classes (escitalopram, sertraline, and venlafaxine), although there was no placebo treatment^52^. In the EMBARC placebo-controlled trial, higher connectivity within the DMN as well as between the DMN and executive control networks predicted better outcomes specifically for sertraline. From a seed-based connectivity analysis, low functional connectivity in the dorsolateral prefrontal cortex and subcallosal cingulate cortex and high connectivity in the ventral striatum and amygdala were associated with a greater improvement from the antidepressant medication sertraline relative to placebo^53^.

Our findings reveal that medication-free first-episode and recurrent MDD are characterized by two neuroanatomical dimensions that suggest distinct responses to SSRI antidepressant medications and placebo. D1 showed a significantly greater clinical improvement with SSRI antidepressant medication relative to placebo, whereas D2 showed no significant differences in treatment effects between SSRI antidepressants and placebo. Antidepressant medications demonstrate significantly greater treatment efficacy than placebo in randomized controlled MDD trials^54,55^. The effects are clinically significant with greater symptom severity, as defined by the UK National Institute of Health and Social Care. How measures of treatment efficacy translate into a clinically meaningful benefit has important implications at the individual level^56^. Moreover, receiving placebo treatment as part of a clinical trial involves systematic follow-up visits, which is not the same as receiving ‘no treatment’^57^.

Yet it is not possible to predict treatment response to any antidepressant medication or to placebo. We found that D1 shows distinct responses to SSRIs and placebo in MDD participants in a current episode of moderate severity. The present findings support the possibility of identifying at the individual level MDD participants who will show a greater likelihood of treatment response to SSRI antidepressant medication relative to placebo. Choosing the right treatment would lead to earlier improvements in depression symptoms and reduce morbidity associated with persistent symptoms. The dimensions reveal a potential neuroimaging-based marker that can predict treatment outcome to SSRI and placebo, offering an important step toward treatment stratification.

Limitations of the present study include the lack of repeated longitudinal MRI measures for each treatment arm. The analysis was focused on the baseline measurements during a current depression episode, limiting the analysis to depressive state rather than as a trait-like feature. Macroscopic structural abnormalities have been linked with microstructural cytoarchitectonic properties^58^. How neuroanatomy might change following treatment and effects on the observed dimensions is unclear but will be examined in the studies that have acquired repeated MRI scans. The present analysis was limited to a single modality; preliminary functional connectivity measures indicate that there are additional dimensions^59^. Surface and thickness indices are genetically independent, potentially providing distinct contributions to treatment response predictions^60^. Functional connectivity in combination with neuroanatomical dimensions has the potential to yield a novel neuroanatomical–neurofunctional coordinate system^28^. As previous history of antidepressant medication treatment has been associated with a greater response to antidepressant medication relative to placebo^61^, it is possible that medication history might distinguish the two dimensions. Of note, we did not include treatment-resistant depression, which is characterized by a history of multiple serial treatment trials and often combination of treatments. The present findings were fully data-driven, and it is not possible to predict treatment response at the individual patient level solely on the basis of treatment history. Nonetheless, the findings might reflect previous antidepressant use or the neurobiological impact of other clinical factors, which are not clinically predictive at the individual patient level.

In summary, MDD is a heterogeneous disorder with widespread subtle neuroanatomical correlates. In the present study, we used a semi-supervised clustering method in a large multisite sample consisting of deeply phenotyped, medication-free MDD individuals in a current depression episode. We found two neuroanatomical dimensions that showed distinct treatment responses to SSRI medications and to placebo. D1 demonstrated preserved volumes and showed greater clinical improvements with SSRI antidepressant medication relative to placebo, while D2 was associated with widespread reduced volumes and no significant difference in treatment responses to either SSRIs or placebo. The present findings indicate that MDD is composed of neuroanatomical dimensions that have distinct treatment responses, offering the potential to develop neuroimaging-based markers in combination with other markers for disease identification and prediction of treatment response.

## Methods

### Participants

COORDINATE-MDD is an international consortium consisting of raw individual MRI data with deep phenotypic characterization in MDD^28^. Ethical approvals were acquired by institutional review boards for each study site. The subset of MDD participants included in the present study satisfied the following inclusion criteria: (1) *Diagnostic and Statistical Manual of Mental Disorders* 4th Edition (*DSM-IV*) based diagnosis of MDD; (2) in current depression episode of at least moderate severity, defined as a 17-item Hamilton Rating Scale for Depression score equal to or greater than 14; (3) medication free at the time of scanning. Exclusion criteria were as follows: (1) current comorbid psychiatric, medical, or neurological disorders; (2) treatment-resistant depression, defined as not achieving clinical response to two or more trials of antidepressant medications. A flowchart depicting the screening process is in Supplementary Fig. 3.

The present study consists of a total of 685 MDD participants from 10 studies (datasets are described in detail in the Supplementary Information): CAN-BIND^62^ (*N* = 92), EMBARC^63^ (*N* = 257), Huaxi MR Research Center SCU (HMRRC^64^, *N* = 111), King’s College London (KCL^65^, *N* = 20), Manchester Remedi^66,67^ (*N* = 40), Laureate Institute for Brain Research (LIBR^68,69^, *N* = 554), Oxford^70^ (*N* = 39), Predictors of Remission in Depression to Individual and Combined Treatments (PReDICT^71^, *N* = 63), Stanford SNAP^72^ (*N* = 8), and Stratifying Resilience and Depression Longitudinally (STRADL^73^, *N* = 1); and a total of 699 healthy control (HC) participants from 10 studies: CAN-BIND (*N* = 23), EMBARC (*N* = 39), KCL (*N* = 20), LIBR (*N* = 141), Manchester Blame (*N* = 46), Manchester Remedi (*N* = 30), Oxford (*N* = 31), HMRRC SCU (*N* = 139), Stanford SNAP (*N* = 50), and STRADL (*N* = 180). EMBARC is a publicly available dataset. All other data were shared and aggregated through the COORDINATE-MDD consortium^28^. We obtained anonymized demographic, clinical, and MRI data from the principal investigators of the original studies that contributed to the present analysis. The data were acquired under a data-sharing agreement that allows us to access and analyze the data as collaborators in the consortium. The data do not contain any information that could identify the participants in the original studies.

The pooled age range was 18–65 years for MDD and 16–72 years for healthy control participants. MDD diagnosis was based on *DSM-IV* or *DSM-IV* Text Revision diagnostic criteria. The number of MDD participants who were treatment-naïve is 128. Information about ethnicity (self-reported) can be found in Table 1. Missing information is because data either were not collected or were not shared. Image protocols, scanner acquisition parameters, and study characteristics can be found in Table 1 and Supplementary Information. Demographic information by site, for patients and controls, can be found in Supplementary Tables 2 and 3. Each study was approved by the local ethics committee, and all participants gave written consent to participate and share de-identified data according to each institution’s local legislative and/or ethical policies. Ethical approval numbers are as follows: Manchester (Stockport Research Ethics Committee 07/H1012/76), SNAP (IRB approval 12104), EMBARC (STU 092010–151), Oxford (REC reference 11/SC/0224), LIBR (WCG IRB 1136261 and 1136947), STRADL (NHS Tayside committee 14/SS/0039), PReDICT (Emory IRB # 00024975), KCL (Bromley NHS REC 13/LO/0904), and SCU (IRB 2020(54)).

Longitudinal treatment outcomes were available in a subset of five prospective clinical treatment trials: CAN-BIND (*N* = 81), EMBARC (*N* = 207), Oxford (*N* = 35), Manchester (*N* = 36), and PReDICT (*N* = 63). The treatments were an SSRI antidepressant medication (citalopram (Manchester), escitalopram (CAN-BIND, Oxford, PReDICT), or sertraline (EMBARC)), an SNRI medication (duloxetine (PReDICT), placebo (EMBARC), or cognitive behavioral therapy (PReDICT). Treatment duration was 6 weeks (Oxford), 8 weeks (CAN-BIND, EMBARC, Manchester), or 12 weeks (PReDICT). Depression symptom severity was assessed by clinician-rated scales: 17-item HAM-D (EMBARC, Oxford, PReDICT)^74^ and Montgomery–Åsberg Depressive Ratings Scale (CAN-BIND, Manchester)^75^. Montgomery–Åsberg ratings were converted into HAM-D rating using conversion tables^76^. Symptom ratings were acquired at baseline and following treatment for all studies (Table 1). Trial registration numbers are as follows: CAN-BIND (NCT01655706), EMBARC (NCT01407094), and PReDICT (NCT00360399). Oxford and Manchester do not have clinical trial registration because it was not a national or funder requirement at the time.

### Image preprocessing

Each participant’s quality-controlled T1-weighted MRI image was preprocessed with a containerized processing pipeline. Preprocessing steps consisted of correction for magnetic field intensity inhomogeneity followed by multi-atlas skull-stripping^77^. Images were segmented using a state-of-the-art multi-atlas, label fusion method (MUSE) to derive 259 pre-defined anatomical regions of interest (ROIs) of the segmented tissue maps^19^ (the list of ROIs can be found in Supplementary Table 4). Voxel-wise regional volumetric maps (RAVENS) were generated for each tissue volume^78^ by spatially aligning the skull-stripped images to a template in the Montreal Neurological Institute coordinate-space using a registration method^79^ and harmonizing for site, age, and sex effects^80^.

### Application of HYDRA to identify neuroanatomical dimensions

HYDRA is a nonlinear semi-supervised machine-learning clustering method to distinguish patients from controls by combining multiple linear classifiers, whereby each hyperplane separates a dimension of patients from the control group resulting in a ‘1-to-*k*’ mapping^29^. Therefore, HYDRA clusters disease effects by comparing brain patterns with those of healthy controls rather than by comparing patients with one another. The Adjusted Rand Index (ARI) is a measure of similarity between iterations of the clustering process. The Rand Index is the sum of the number of pairs of participants that are clustered in the same subtype in two separate iterations and the number of pairs of participants that are clustered in different subtypes in both iterations, divided by the total possible number of pairs. The ARI is the Rand Index adjusted for chance such that the upper bound ARI = 1 indicates that all participants are clustered identically across iterations whereas an ARI = 0 indicates that participants are randomly assigned into clusters. The ARI is used to identify the optimal number of dimensions (*k*) from a range between 2 and 5. Since HYDRA is a multivariate method, we applied it to the raw MUSE ROIs. To evaluate the robustness of the optimal *k* clusters scheme, we performed additional analyses. First, we used split-sample analyses to evaluate the robustness of the optimal *k* dimension solution to assess whether the dimensions in each half exhibit similar neuroanatomical patterns, given that the two halves have similar cohort characteristics in terms of age, sex, and site. Second, we conducted leave-site-out cross-validation to examine whether the dimensions were being driven by any one particular site.

### Voxel-wise RAVENS of regional tissue volumes

Voxel-wise RAVENS gray- and white-matter maps^78^ were used to identify the brain regions that differentiate each HYDRA dimension from the healthy control group. Statistical parametric maps estimating deviations from healthy controls for each dimension were calculated using regionally linear multivariate discriminative statistical mapping^81^ with age and sex as covariates and filtering out non-significant voxels (p_FDR_ < 0.05). Covariate effects were first removed from the data using a linear model and then the core method for detecting group differences was run for the remaining variable of interest (patients versus controls). For completeness, we examined the gray-matter differences between the MDD participant group as a whole and healthy controls while controlling for age, sex, and years of education. In a second model, we also controlled for medication history as an additional covariate. Medication history, which was measured by the number of antidepressant medication trials, was available for only one site (CAN-BIND). Since we did not have individual medication information for the rest of the sample, we used a proxy measure as an estimate of previous medication use. MDD participants in a first episode of depression were medication-naïve and would not have taken previous antidepressant medications, whereas MDD participants with recurrent depression would have. Last, to better understand the regional gray-matter differences in first-episode MDD participants relative to healthy controls, we excluded the MDD participants with recurrent depression (all other covariates remained the same). Regions have been labeled with reference to the MUSE atlas^19^. HYDRA and all voxel-wise analyses were performed in MATLAB 2018A.

### Statistics

#### Demographic and clinical variables

Group comparisons for demographic (age, sex, and years of education) and clinical (age of onset, years of illness, and duration of current episode in weeks) variables were examined across the HYDRA dimensions using Mann–Whitney U tests for continuous variables (for example, age) and chi-square tests for categorical variables (for example, sex).

#### Evaluation of HYDRA dimensions and their treatment response to antidepressant and placebo

The subset consisted of four cohorts of MDD participants from the prospective, longitudinal clinical treatment trials that had included healthy control participants from the same sites: CAN-BIND (*N* = 81), EMBARC (*N* = 207), Oxford (*N* = 35), and Manchester (*N* = 36). Treatment was SSRI antidepressant (citalopram (Manchester), escitalopram (CAN-BIND, Oxford), or sertraline (EMBARC)) or placebo (EMBARC). Treatment duration was six weeks (Oxford) or eight weeks (CAN-BIND, EMBARC, and Manchester).

Of the five cohorts with longitudinal treatment outcomes, PReDICT (*N* = 63) had included only MDD participants. As robustness of the optimal dimensional clustering involves comparison of the patterns between patients and healthy controls, we could not be certain about the results for the five cohorts; therefore, we present the results for four cohorts here, and the results including PReDICT are presented in Supplementary Figs. 4 and 5.

To examine interactions between HYDRA dimension and treatment group, we used a linear regression model with the percentage change in the clinician-rated depressive symptom scale (continuous) as the outcome variable and HYDRA dimension (categorical, two groups) and treatment group (categorical, two groups: SSRI and placebo) as the independent variables while controlling for age, sex, and site. Percentage change in score was calculated as follows: (pre-treatment baseline score – post-treatment score)/pre-treatment score × 100. The effect size (Cohen’s *f*^2^ = 0.06) of the interaction term has an *F* statistic of 3.607 on the basis of our analysis using a linear regression model. With a sample size of 359, assuming that we adjust for six additional covariates in the model and the same effect size, we have over 99% power to detect a significant interaction term between treatment and HYDRA dimension under 5% Type I error. We chose *P* = 0.05 (two-sided) as the threshold for significance. The analyses were repeated while controlling for additional confounding factors (years of education and medication status) and are presented in Supplementary Results 2.

The linear regression models were conducted using the statsmodels 0.13.1 Python module^82^. Power analyses, Mann–Whitney U tests, and chi-square tests were conducted in R version 4.2.2.

In a machine-learning analysis, we trained a support vector machine to classify patients between the identified HYDRA dimensions and performed an additional linear regression using the calculated hyperplane distance in place of the dimension label.

### Reporting summary

Further information on research design is available in the Nature Portfolio Reporting Summary linked to this article.

### Supplementary information

Supplementary Information Supplementary Figs. 1–4, Tables 1–5, Methods 1 and 2, and Results 1 and 2.

Reporting Summary

## Data Availability

CAN-BIND data are available from https://www.braincode.ca/; EMBARC data are available from https://nda.nih.gov/edit_collection.html?id=2199; original data are available from individual co-authors, and the derived data are available on reasonable request to corresponding authors C.H.Y.F. and C.D.

## Code availability

The MUSE algorithm for image segmentation is available at https://www.nitrc.org/projects/cbica_muse. The HYDRA algorithm is available at https://github.com/evarol/HYDRA. The MIDAS algorithm is available at https://github.com/evarol/MIDAS. The following R packages were used: WebPower 0.8.6 (https://cran.r-project.org/web/packages/WebPower/WebPower.pdf), effectsize 0.8.2, and ggplot2 3.4.0. (https://cran.r-project.org/web/packages/ggplot2/ggplot2.pdf).
