# Supplementary material for: Neuroanatomical dimensions in medication-free individuals with major depressive disorder and treatment response to SSRI antidepressant medications or placebo
Source: Nat Ment Health. 2024 Jan 12;2(2):164–76. doi: 10.1038/s44220-023-00187-w (PMC11211072; doi:10.1038/s44220-023-00187-w)
Supplement: Supplementary file 1 — Supplementary Figs. 1–4, Tables 1–5, Methods 1 and 2, and Results 1 and 2. [file 44220_2023_187_MOESM1_ESM.pdf]

nature mental health

Article https://doi.org/10.1038/s44220-023-00187-w

Neuroanatomical dimensions in medication-free individuals with major depressive disorder and treatment response to SSRI antidepressant medications or placebo

In the format provided by the authors and unedited

Supplementary materials

Neuroanatomical dimensions in medication-free individuals with major depressive disorder and treatment response to SSRI antidepressant medications or placebo

Cynthia H.Y. Fu, Mathilde Antoniades, Guray Erus, Jose A. Garcia, Yong Fan, Danilo Arnone, Stephen R. Arnott, Taolin Chen, Ki Sueng Choi, Cherise Chin Fatt, Benicio N. Frey, Vibe G. Frokjaer, Melanie Ganz, Beata R. Godlewska, Stefanie Hassel, Keith Ho, Andrew M. McIntosh, Kun Qin, Susan Rotzinger, Matthew D. Sacchet, Jonathan Savitz, Haochang Shou, Ashish Singh, Aleks Stolicyn, Irina Strigo, Stephen C. Strother, Duygu Tosun, Teresa A. Victor, Dongtao Wei, Toby Wise, Roland Zahn, Ian M. Anderson, W. Edward Craighead, J.F. William Deakin, Boadie W. Dunlop, Rebecca Elliott, Qiyong Gong, Ian H. Gotlib, Catherine Harmer, Sidney H. Kennedy, Gitte M. Knudsen, Helen S. Mayberg, Martin P. Paulus, Jiang Qiu, Madhukar H. Trivedi, Heather C. Whalley, Chao-Gan Yan, Allan H. Young, Christos Davatzikos

Table of Contents

Supplementary Method 1. Description of main datasets in this study 3
Supplementary Method 2. Estimation of years of education 5
Supplementary Results 1. Comparison of estimated prior medication status across HYDRA dimensions 6
Supplementary Results 2. Interaction between HYDRA dimensions and treatment outcomes whilst controlling for medication and education 7
Supplementary Figure 1 8
Supplementary Figure 2 9
Supplementary Figure 3 10
Supplementary Figure 4 11
Supplementary Figure 5 12
Supplementary Table 1. Scanner protocols 13
Supplementary Table 2. Demographic information for healthy control participants by site 14
Supplementary Table 3. Demographic information for MDD participants by site 15
Supplementary Table 4. List of structural MUSE regions of interest used in the HYDRA model 16
Supplementary Table 5. Regional volumetric differences between patients with first-episode depression and healthy controls. 23
Supplementary References 27

Supplementary Method 1. Description of main datasets in this study

Full descriptions of the datasets are available in: Fu CHY, Erus G, Fan Y, Antoniades M, et al., AI-based dimensional neuroimaging system for characterizing heterogeneity in brain structure and function in major depressive disorder: COORDINATE-MDD consortium design and rationale. BMC Psychiatry. Jan 23 2023;23(1):59. doi:10.1186/s12888-022-04509-7.

Main datasets in this study are as follows:

1. Canadian Biomarker Integration Network in Depression (CAN-BIND) is a national depression program with recruitment from 7 centers (MacQueen et al., 2019). Treatment protocol is 8-week trial with SSRI antidepressant (escitalopram) followed by an augmentation trial if there is poor treatment response (i.e., less than 50% improvement in depressive symptoms). MRI scans have been acquired at baseline, weeks 2 and 8 in both MDD and healthy participants.

2. Establishing Moderators and Biosignatures of Antidepressant Response in Clinical Care (EMBARC) is a multisite, randomized, placebo-controlled clinical trial with recruitment from 4 centers (Trivedi et al., 2016). Treatment protocol is an 8-week double-blind randomized allocation to SSRI (escitalopram) or placebo, with double-blind cross over switch to another antidepressant if there is poor treatment response. MRI scans have been acquired in MDD and healthy participants at baseline.

3. Huaxi MR Research Center at Sichuan University (SCU) cohort consists of medication-naïve first episode MDD and matched healthy participants (Qiu et al., 2018; Zhao et al., 2020; Zhao et al., 2021).

4. King's College London cohort consists of 4 studies (Green et al., 2012; Nouretdinov et al., 2011; Sankar et al., 2016; Wise et al., 2017, 2018). MRI scans have been acquired in MDD and healthy participants, and the treatment study is an 8-week selective serotonin and norepinephrine reuptake inhibitor (SNRI) antidepressant (duloxetine) with MRI scans at baseline, weeks 2 and 8 in both MDD and healthy participants.

5. Laureate Institute for Brain Research (LIBR) cohort consists of MRI data in first episode and recurrent MDD and matched healthy controls from 2 studies (Ford et al., 2019; Misaki et al., 2016; Zheng et al., 2021).

6. Manchester cohort consists of 3 studies (Arnone et al., 2012, 2013; Dutta et al., 2019). All participants have a baseline MRI scan, and the treatment study is 8-week SSRI (citalopram) with MRI scans at baseline and week 8 (Arnone et al., 2012).

7. Oxford cohort consists of 6-week SSRI (escitalopram) treatment with MRI scans at baseline and week 6 (Godlewska et al., 2014, 2018).

8. Predictors of Remission in Depression to Individual and Combined Treatments (PReDICT) study is a 12-week randomized clinical trial of treatment-naïve MDD participants with 3 treatment arms: SSRI (escitalopram), SNRI (duloxetine), or CBT, with an augmentation trial if is there no remission. MRI scans were acquired at baseline (Dunlop et al., 2012).

9. Southwest University (SWU) cohort consists of a community-based recruitment which includes first episode and recurrent MDD and healthy control participant (Liu et al., 2017, 2021; Hu et al., 2021).

10. Stanford (SNAP) cohort consists of first episode and recurrent MDD and healthy participants (Sacchet & Gotlib, 2017; Sacchet et al., 2019).

11. Stratifying Resilience and Depression Longitudinally (STRADL) is a community-based cohort from the Generation Scotland Scottish Family Health Study with detailed clinical, cognitive and neuroimaging assessments (Habota et al., 2019). Single session MRI scans were acquired.

All patients are adults with a primary diagnosis of MDD that is first episode or recurrent, in a current episode of a moderate to severe severity, that is non-psychotic. MDD diagnosis was based on DSM-IV (SCU, Manchester, Oxford) or DSM-IV-TR (CAN-BIND, EMBARC, KCL, LIBR, Stanford SNAP, STRADL) using the Structured Clinical Interview for DSM (SCID) (EMBARC, SCU, KCL, Manchester, Oxford, Stanford SNAP, STRADL) or Mini International Neuropsychiatric Interview (MINI) (CAN-BIND, KCL).

Depressive severity was measured with standardized clinician-rated scales: 17-item Hamilton Rating Scale for Depression (HRSD, (Hamilton, 1960) (EMBARC, SCU, KCL, Oxford, PREDICT, Stanford SNAP), Montgomery-Åsberg Depression Rating Scale (MADRS) (Montgomery & Asberg, 1979)(CAN-BIND, LIBR, Manchester), or Quick Inventory of Depressive Symptomatology (QIDS) (Rush et al., 2003; Trivedi et al., 2004)(STRADL). The rating scales show high correlation in summed scores and percentage score improvements (Leucht et al., 2018; Rush et al., 2003; Uher et al., 2008).

Supplementary Method 2. Estimation of years of education

Wherever raw text was provided, it was converted into years of education. Unless indicated we assumed that the degree was completed and allocated the maximum number of years associated with completing the degree or diploma. The calculation assumed that participants started school at the age of 5.

Raw text	Years of education attributed
No education	0
Primary school	6
Middle school	8
Some high school (10th, 11th)	11
High school	12
GED or alternative credential	12
12th grade, no diploma	12
Some college credit, but less than 1 year of college credit	12.5
1 or more years of college credit, no degree	13
Some college, no degree	13
Some college	14
Tech school	14
Associate's degree (for example: AA, AS)	14
Trade school	14
College and above	16
Four-year college	16
Junior College	16
Bachelor's degree	16
Some graduate school	17
Grad or Prof Degree	18
Master's degree	18
Doctorate	23

Supplementary Results 1. Comparison of estimated prior medication status across HYDRA dimensions

In order to compare medication exposure across HYDRA dimensions we used a Chi-squared test to determine if the dimensions differed in the number of MDD participants with first episode or recurrent depression. In Dimension 1, 201 participants had recurrent MDD and 89 participants had first episode MDD. In D2, 222 patients had recurrent MDD and 173 had first episode MDD. The Chi-squared test showed a significant difference between the dimensions (X^2=11.6, p=0.0007).

Supplementary Results 2. Interaction between HYDRA dimensions and treatment outcomes whilst controlling for medication and education

The interaction between HYDRA dimension and treatment group was examined using a linear regression model with the percentage change in the clinician-rated depressive symptom scale (continuous) as the outcome variable and HYDRA dimension (categorical, 2 groups) and treatment group (categorical, 2 groups: SSRI and placebo) as the independent variables whilst controlling for age, sex, site, years of education (as a proxy for IQ) and medication status (using first-episode or recurrent MDD as a proxy measure).

Treatment with SSRI medications was associated with a significantly greater improvement in depressive symptoms across both D1 and D2 (β=34.5, 95% CI (7.2 to 61.7), p=0.01).

The Dimension by treatment interaction remained significant after controlling for age, sex, site, medication status and years of education (β=-19.1, 95% CI (-36.4 to -1.8), p=0.03).

In order to examine whether the interaction between Dimensions and treatment group differed according to SSRI medication, we performed a second linear regression with the treatment group variable including all four treatment categories (SSRI sertraline, SSRI escitalopram, SSRI citalopram and placebo) instead of a binary category (SSRI medications and placebo). The covariates of this linear model included age, sex, site, medication status and years of education. There was a significant interaction between treatment with sertraline and HYDRA dimension (β=-24.1, 95% CI (-43.8 to -4.4), p=0.02). The interaction was not significant for escitalopram (β=-12.1, 95% CI (-33.8 to 9.6), p=0.27) or citalopram (β=5.9×10^{-15}, 95% CI (-1.3×10^{-15} to 1.3×10^{-14}), p=0.11).

Supplementary Figure 1

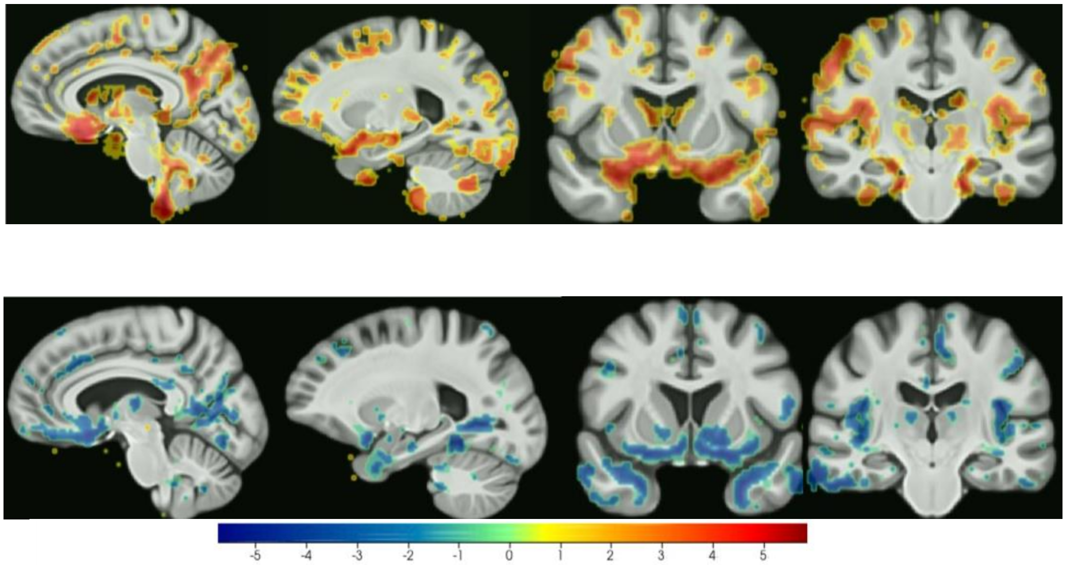

Additional coronal and sagittal views for dimensions 1 and 2 (top and bottom rows respectively), indicating areas with significant differences from controls. Colour represents direction and strength of group differences as indicated by the colour bar.

Supplementary Figure 2

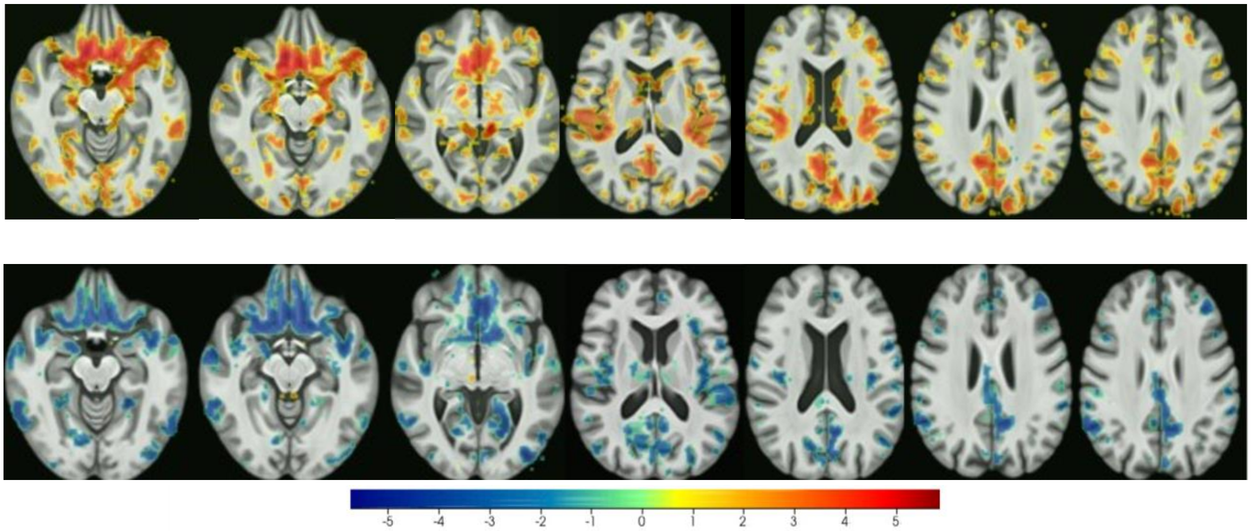

Additional transverse views for dimensions 1 and 2 (top and bottom rows respectively), indicating areas with significant differences from controls. Colour represents direction and strength of group differences as indicated by the colour bar.

Supplementary Figure 3

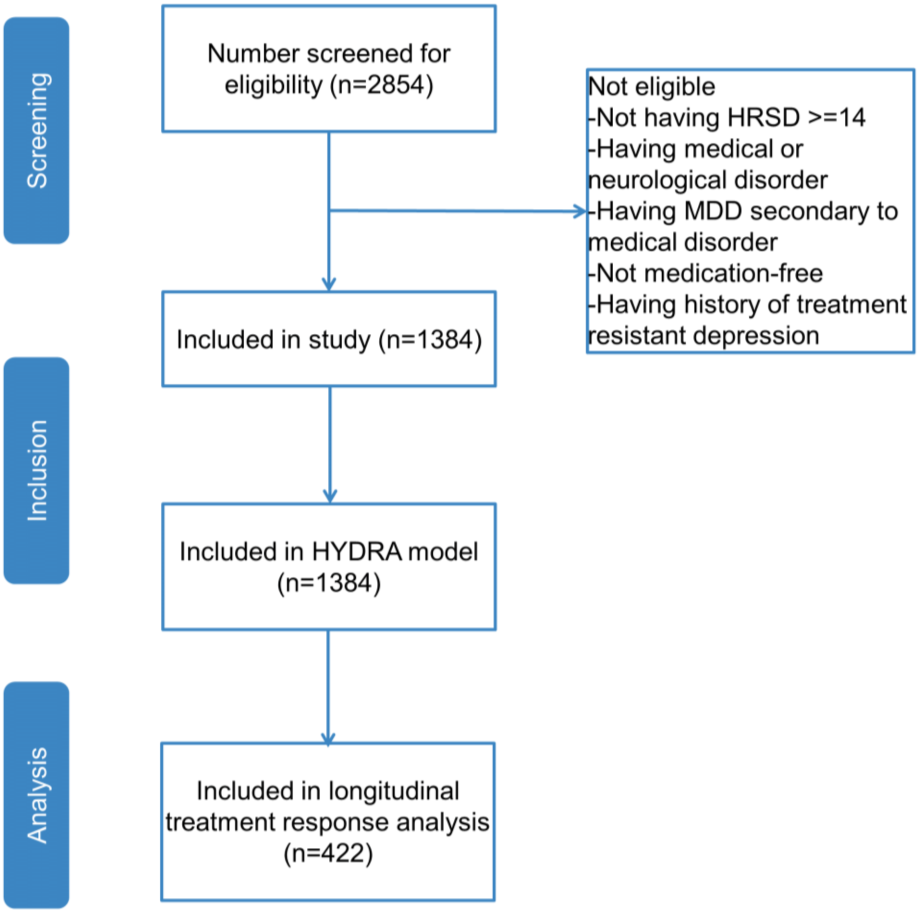

Supplementary Figure 3. Flow diagram of the screening process to include subjects in the final analyses.

Supplementary Figure 4

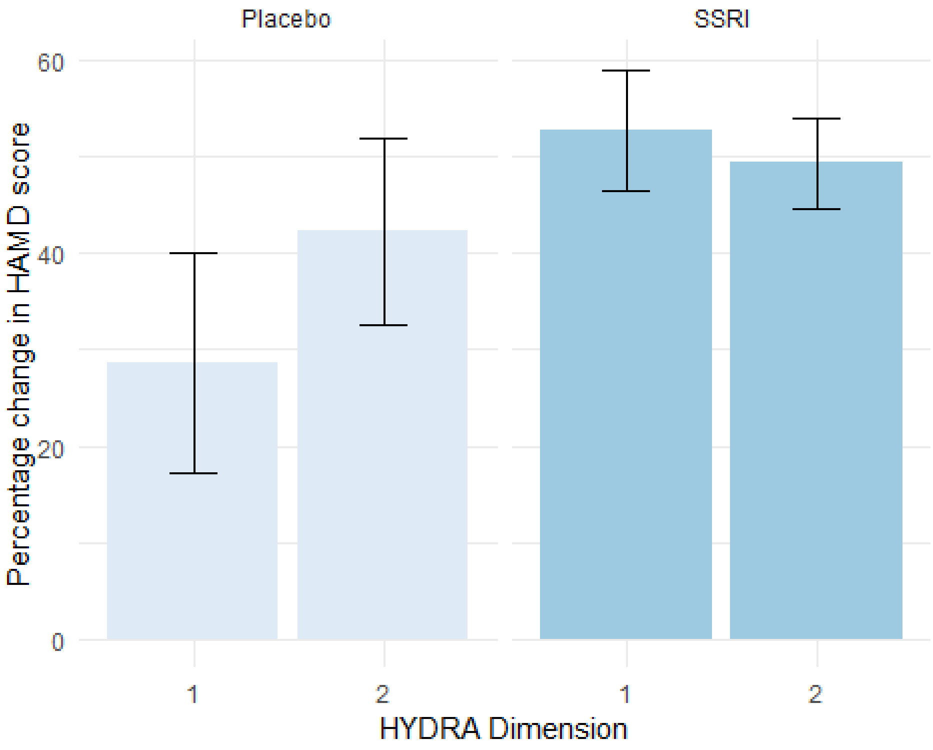

Supplementary Figure 4. Difference in percentage change in HAMD scores across HYDRA Dimensions (D1 (n=169) and D2 (n=253), n=422) and binary treatment groups following treatment with SSRI medications (n=313) and placebo (n=109). The sample consists of all five cohorts including PReDICT. Data are presented using a bar plot as mean values and 95th percentile error bars. The asterisks (*) indicate significant differences between the two subgroups using linear regression model (two-sided $P < 0.05$).

Supplementary Figure 5

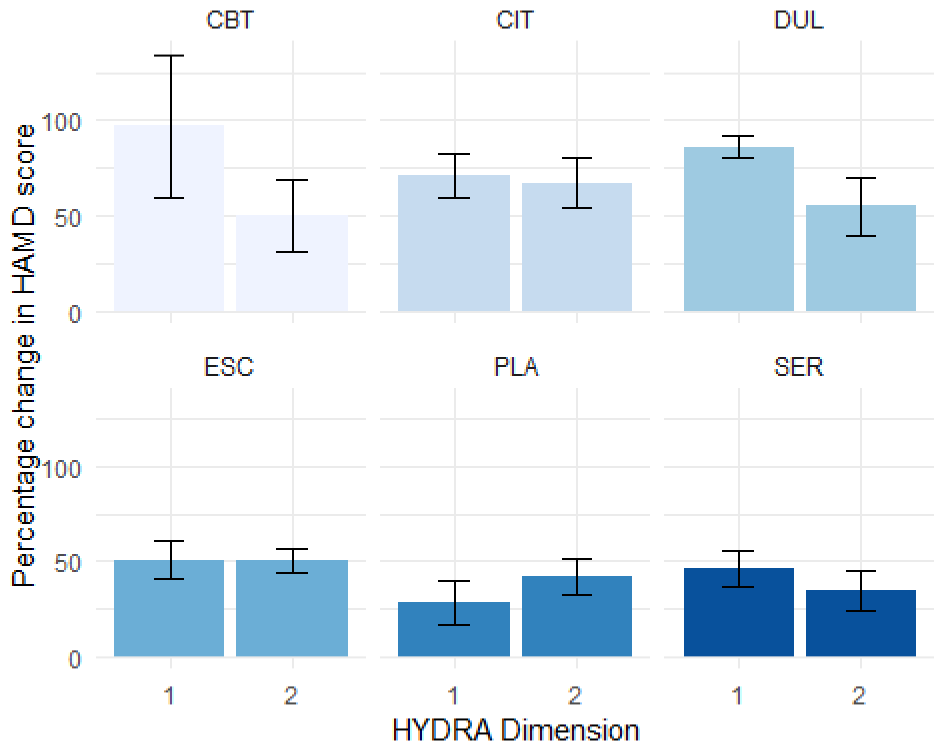

Supplementary Figure 5. Difference in percentage change in HAMD scores across HYDRA Dimensions (D1 (n=164) and D2 (n=195), n=359) and 6 different treatment groups following treatment with cognitive behavioral therapy (CBT, N=19), placebo medication (PLA, N=109), citalopram medication (CIT, n=36), duloxetine medication (DUL, N=20), escitalopram medication (ESC, N=140), sertraline medication (SER, n=98). The sample consists of all five cohorts including PReDICT. Data are presented using a bar plot as mean values and 95th percentile error bars. The asterisks (*) indicate significant differences between the two subgroups using linear regression model (two-sided $P < 0.05$).

Supplementary Table 1. Scanner protocols

	CAN-BIND	EMBARC	HMRRC	Manchester	LIBR	Oxford	PReDICT	Stanford	STRADL
**Scanner model**	GE 3T Signa HDxt	All 3T: GE Signa HDx; Siemens TrioTim, Philips Ingenia	3T Siemens Trio	Philips Intera 1.5T	GE 3T Discovery MR750	3T Siemens TIM Trio	3T Siemens Magnetom TIM Trio	1.5T GE Signa Excite	3T Philips Achieva TX; Siemens 3T Prisma fit
**T1 Resolution (mm^3)**	1x1x1	1x1x1	0.94x0.94x1	0.875x0.875x1	0.9375x0.9375x0.9	1x1x1	1x1x1	0.86x0.86	1x1x1
**TR/TE (ms)**	7.5/2.86	5.9-8.2/2.4-3.7	1900/2.26	8.99/4.2	5.0/2.0	20040/ 4.68	2300/2-4	8.3-10.1/ 1.7-3.0	8.2/3.8
**Number of volumes**	176	160	176	160	120			116	160

Supplementary Table 2. Demographic information for healthy control participants by site

Site	Sample size	Age (mean)	F/M (n)	Years of education (mean)	HAMD	MADRS	QIDS
**CAN-BIND**	23	33.3	12/11	15.1		0.6	
**EMBARC**	39	37.1	24/15	15.1	0.6		
**HMRRC**	139	30.9	71/68	12.9	NA		
**KCL-Blame**	46	33.4	29/17	17.3		0.7	
**KCL-BUD**	20	30.1	18/2	17.5		1.0	
**LIBR-B**	81	30.5	44/37	15.0	1.9		
**LIBR-S**	60	32.0	39/21	14.6	1.8		
**Manchester**	30	33.3	21/9	NA		0.1	
**Oxford**	31	30.3	18/13	NA	0.4		
**PReDICT**	0	NA	NA	NA			
**Stanford**	50	32.6	33/17	16.0	2.0		
**STRADL**	180	56.8	95/85	16.0			2.7
**Total**	699	38.4	404/295	15.3	1.5	0.6	2.7

Age was not part of our inclusion/exclusion criteria so it was just happenstance that the groups have different age ranges. The number of controls by age above 65 is as follows:

**65**	N=6
**66**	N=4
**67**	N=2
**68**	N=4
**69**	N=1
**70**	N=2
**71**	N=4
**72**	N=1

Supplementary Table 3. Demographic information for MDD participants by site

Site	Sample size	Dimension 1/2	Age (mean)	F/M (n)	FE/RC	Years of education (mean)	Age of onset (mean, sd)	HAMD	MADRS	QIDS
**CAN-BIND**	92	27/65	36.0	61/31	14/49	13.9	22.0 (10.9)		29.7	
**EMBARC**	257	134/123	36.6	166/91	1/253	15.1		19.5		
**HMRRC**	111	53/58	31.7	65/46	81/0	13.2		25.2		
**KCL-BUD**	20	10/10	29.6	18/2		15.1	22.8 (7.8)		27.3	
**LIBR-B**	32	14/18	35.3	21/11		14.0		18.5		
**LIBR-S**	22	12/10	35.2	15/7		14.0		19.6		
**Manchester**	40	19/21	36.4	27/13	3/37		22.0 (8.1)		27.3	
**Oxford**	39	14/25	29.9	24/15	25/14		25.4 (9.1)	22.7		
**PReDICT**	63	5/58	39.3	36/27		15.0	29.7 (12.2)	18.3		
**Stanford**	8	2/6	34.0	5/3	4/2	15.0		17.5		
**STRADL**	1	0/1	59.0	1/0		16.0				16
**Total**	685	290/395	35.3	439/246	128/355	14.5	24.5 (10.8)	21.2	29.0	16

Supplementary Table 4. List of structural MUSE regions of interest used in the HYDRA model

ROI index	ROI Name	ROI Resolution
**4**	3rd Ventricle	Single
**11**	4th Ventricle	Single
**23**	Right Accumbens Area	Single
**30**	Left Accumbens Area	Single
**31**	Right Amygdala	Single
**32**	Left Amygdala	Single
**35**	Brain Stem	Single
**36**	Right Caudate	Single
**37**	Left Caudate	Single
**38**	Right Cerebellum Exterior	Single
**39**	Left Cerebellum Exterior	Single
**40**	Right Cerebellum White Matter	Single
**41**	Left Cerebellum White Matter	Single
**47**	Right Hippocampus	Single
**48**	Left Hippocampus	Single
**49**	Right Inferior Lateral Ventricle	Single
**50**	Left Inferior Lateral Ventricle	Single
**51**	Right Lateral Ventricle	Single
**52**	Left Lateral Ventricle	Single
**55**	Right Pallidum	Single
**56**	Left Pallidum	Single
**57**	Right Putamen	Single
**58**	Left Putamen	Single
**59**	Right Thalamus Proper	Single
**60**	Left Thalamus Proper	Single
**61**	Right Ventral DC	Single
**62**	Left Ventral DC	Single
**71**	Cerebellar Vermal Lobules I-V	Single
**72**	Cerebellar Vermal Lobules VI-VII	Single
**73**	Cerebellar Vermal Lobules VIII-X	Single
**75**	Left Basal Forebrain	Single
**76**	Right Basal Forebrain	Single
**81**	Frontal lobe white matter right	Single
**82**	Frontal lobe white matter left	Single
**83**	Occipital lobe white matter right	Single
**84**	Occipital lobe white matter left	Single
**85**	Parietal lobe white matter right	Single
**86**	Parietal lobe white matter left	Single
**87**	Temporal lobe white matter right	Single

**88**	Temporal lobe white matter left	Single
**89**	Right fornix	Single
**90**	Left fornix	Single
**91**	Anterior limb of internal capsule right	Single
**92**	Anterior limb of internal capsule left	Single
**93**	Posterior limb of internal capsule inc. cerebral peduncle right	Single
**94**	Posterior limb of internal capsule inc. cerebral peduncle left	Single
**95**	Corpus callosum	Single
**100**	Right anterior cingulate gyrus	Single
**101**	Left anterior cingulate gyrus	Single
**102**	Right anterior insula	Single
**103**	Left anterior insula	Single
**104**	Right anterior orbital gyrus	Single
**105**	Left anterior orbital gyrus	Single
**106**	Right angular gyrus	Single
**107**	Left angular gyrus	Single
**108**	Right calcarine cortex	Single
**109**	Left calcarine cortex	Single
**112**	Right central operculum	Single
**113**	Left central operculum	Single
**114**	Right cuneus	Single
**115**	Left cuneus	Single
**116**	Right entorhinal area	Single
**117**	Left entorhinal area	Single
**118**	Right frontal operculum	Single
**119**	Left frontal operculum	Single
**120**	Right frontal pole	Single
**121**	Left frontal pole	Single
**122**	Right fusiform gyrus	Single
**123**	Left fusiform gyrus	Single
**124**	Right gyrus rectus	Single
**125**	Left gyrus rectus	Single
**128**	Right inferior occipital gyrus	Single
**129**	Left inferior occipital gyrus	Single
**132**	Right inferior temporal gyrus	Single
**133**	Left inferior temporal gyrus	Single
**134**	Right lingual gyrus	Single
**135**	Left lingual gyrus	Single
**136**	Right lateral orbital gyrus	Single
**137**	Left lateral orbital gyrus	Single
**138**	Right middle cingulate gyrus	Single
**139**	Left middle cingulate gyrus	Single
**140**	Right medial frontal cortex	Single

**141**	Left medial frontal cortex	Single
**142**	Right middle frontal gyrus	Single
**143**	Left middle frontal gyrus	Single
**144**	Right middle occipital gyrus	Single
**145**	Left middle occipital gyrus	Single
**146**	Right medial orbital gyrus	Single
**147**	Left medial orbital gyrus	Single
**148**	Right postcentral gyrus medial segment	Single
**149**	Left postcentral gyrus medial segment	Single
**150**	Right precentral gyrus medial segment	Single
**151**	Left precentral gyrus medial segment	Single
**152**	Right superior frontal gyrus medial segment	Single
**153**	Left superior frontal gyrus medial segment	Single
**154**	Right middle temporal gyrus	Single
**155**	Left middle temporal gyrus	Single
**156**	Right occipital pole	Single
**157**	Left occipital pole	Single
**160**	Right occipital fusiform gyrus	Single
**161**	Left occipital fusiform gyrus	Single
**162**	Right opercular part of the inferior frontal gyrus	Single
**163**	Left opercular part of the inferior frontal gyrus	Single
**164**	Right orbital part of the inferior frontal gyrus	Single
**165**	Left orbital part of the inferior frontal gyrus	Single
**166**	Right posterior cingulate gyrus	Single
**167**	Left posterior cingulate gyrus	Single
**168**	Right precuneus	Single
**169**	Left precuneus	Single
**170**	Right parahippocampal gyrus	Single
**171**	Left parahippocampal gyrus	Single
**172**	Right posterior insula	Single
**173**	Left posterior insula	Single
**174**	Right parietal operculum	Single
**175**	Left parietal operculum	Single
**176**	Right postcentral gyrus	Single
**177**	Left postcentral gyrus	Single
**178**	Right posterior orbital gyrus	Single
**179**	Left posterior orbital gyrus	Single
**180**	Right planum polare	Single
**181**	Left planum polare	Single
**182**	Right precentral gyrus	Single
**183**	Left precentral gyrus	Single
**184**	Right planum temporale	Single
**185**	Left planum temporale	Single

**186**	Right subcallosal area	Single
**187**	Left subcallosal area	Single
**190**	Right superior frontal gyrus	Single
**191**	Left superior frontal gyrus	Single
**192**	Right supplementary motor cortex	Single
**193**	Left supplementary motor cortex	Single
**194**	Right supramarginal gyrus	Single
**195**	Left supramarginal gyrus	Single
**196**	Right superior occipital gyrus	Single
**197**	Left superior occipital gyrus	Single
**198**	Right superior parietal lobule	Single
**199**	Left superior parietal lobule	Single
**200**	Right superior temporal gyrus	Single
**201**	Left superior temporal gyrus	Single
**202**	Right temporal pole	Single
**203**	Left temporal pole	Single
**204**	Right triangular part of the inferior frontal gyrus	Single
**205**	Left triangular part of the inferior frontal gyrus	Single
**206**	Right transverse temporal gyrus	Single
**207**	Left transverse temporal gyrus	Single
**301**	FRONTAL_INFERIOR_GM	Composite
**302**	FRONTAL_INSULAR_GM	Composite
**303**	FRONTAL_LATERAL_GM	Composite
**304**	FRONTAL_MEDIAL_GM	Composite
**305**	FRONTAL_OPERCULAR_GM	Composite
**306**	LIMBIC_CINGULATE_GM	Composite
**307**	LIMBIC_MEDIALTEMPORAL_GM	Composite
**308**	OCCIPITAL_INFERIOR_GM	Composite
**309**	OCCIPITAL_LATERAL_GM	Composite
**310**	OCCIPITAL_MEDIAL_GM	Composite
**311**	PARIETAL_LATERAL_GM	Composite
**312**	PARIETAL_MEDIAL_GM	Composite
**313**	TEMPORAL_INFERIOR_GM	Composite
**314**	TEMPORAL_LATERAL_GM	Composite
**315**	TEMPORAL_SUPRATEMPORAL_GM	Composite
**316**	FRONTAL_INFERIOR_GM_L	Composite
**317**	FRONTAL_INSULAR_GM_L	Composite
**318**	FRONTAL_LATERAL_GM_L	Composite
**319**	FRONTAL_MEDIAL_GM_L	Composite
**320**	FRONTAL_OPERCULAR_GM_L	Composite
**321**	LIMBIC_CINGULATE_GM_L	Composite
**322**	LIMBIC_MEDIALTEMPORAL_GM_L	Composite
**323**	OCCIPITAL_INFERIOR_GM_L	Composite

**324**	OCCIPITAL_LATERAL_GM_L	Composite
**325**	OCCIPITAL_MEDIAL_GM_L	Composite
**326**	PARIETAL_LATERAL_GM_L	Composite
**327**	PARIETAL_MEDIAL_GM_L	Composite
**328**	TEMPORAL_INFERIOR_GM_L	Composite
**329**	TEMPORAL_LATERAL_GM_L	Composite
**330**	TEMPORAL_SUPRATEMPORAL_GM_L	Composite
**331**	FRONTAL_INFERIOR_GM_R	Composite
**332**	FRONTAL_INSULAR_GM_R	Composite
**333**	FRONTAL_LATERAL_GM_R	Composite
**334**	FRONTAL_MEDIAL_GM_R	Composite
**335**	FRONTAL_OPERCULAR_GM_R	Composite
**336**	LIMBIC_CINGULATE_GM_R	Composite
**337**	LIMBIC_MEDIALTEMPORAL_GM_R	Composite
**338**	OCCIPITAL_INFERIOR_GM_R	Composite
**339**	OCCIPITAL_LATERAL_GM_R	Composite
**340**	OCCIPITAL_MEDIAL_GM_R	Composite
**341**	PARIETAL_LATERAL_GM_R	Composite
**342**	PARIETAL_MEDIAL_GM_R	Composite
**343**	TEMPORAL_INFERIOR_GM_R	Composite
**344**	TEMPORAL_LATERAL_GM_R	Composite
**345**	TEMPORAL_SUPRATEMPORAL_GM_R	Composite
**401**	BASAL_GANGLIA	Composite
**402**	DEEP_GM	Composite
**403**	DEEP_WM	Composite
**404**	FRONTAL_GM	Composite
**405**	FRONTAL_WM	Composite
**406**	LIMBIC_GM	Composite
**407**	OCCIPITAL_GM	Composite
**408**	OCCIPITAL_WM	Composite
**409**	PARIETAL_GM	Composite
**410**	PARIETAL_WM	Composite
**411**	TEMPORAL_GM	Composite
**412**	TEMPORAL_WM	Composite
**413**	BASAL_GANGLIA_L	Composite
**414**	DEEP_GM_L	Composite
**415**	DEEP_WM_L	Composite
**416**	FRONTAL_GM_L	Composite
**417**	FRONTAL_WM_L	Composite
**418**	LIMBIC_GM_L	Composite
**419**	OCCIPITAL_GM_L	Composite
**420**	OCCIPITAL_WM_L	Composite
**421**	PARIETAL_GM_L	Composite

**422**	PARIETAL_WM_L	Composite
**423**	TEMPORAL_GM_L	Composite
**424**	TEMPORAL_WM_L	Composite
**425**	BASAL_GANGLIA_R	Composite
**426**	DEEP_GM_R	Composite
**427**	DEEP_WM_R	Composite
**428**	FRONTAL_GM_R	Composite
**429**	FRONTAL_WM_R	Composite
**430**	LIMBIC_GM_R	Composite
**431**	OCCIPITAL_GM_R	Composite
**432**	OCCIPITAL_WM_R	Composite
**433**	PARIETAL_GM_R	Composite
**434**	PARIETAL_WM_R	Composite
**435**	TEMPORAL_GM_R	Composite
**436**	TEMPORAL_WM_R	Composite
**501**	CORPUS_CALLOSUM	Composite
**502**	CEREBELLUM	Composite
**503**	DEEP_WM_GM	Composite
**504**	FRONTAL	Composite
**505**	LIMBIC	Composite
**506**	OCCIPITAL	Composite
**507**	PARIETAL	Composite
**508**	TEMPORAL	Composite
**509**	VENTRICLE	Composite
**510**	CEREBELLUM_L	Composite
**511**	DEEP_WM_GM_L	Composite
**512**	FRONTAL_L	Composite
**513**	LIMBIC_L	Composite
**514**	OCCIPITAL_L	Composite
**515**	PARIETAL_L	Composite
**516**	TEMPORAL_L	Composite
**517**	VENTRICLE_L	Composite
**518**	CEREBELLUM_R	Composite
**519**	DEEP_WM_GM_R	Composite
**520**	FRONTAL_R	Composite
**521**	LIMBIC_R	Composite
**522**	OCCIPITAL_R	Composite
**523**	PARIETAL_R	Composite
**524**	TEMPORAL_R	Composite
**525**	VENTRICLE_R	Composite
**601**	GM	Composite
**604**	WM	Composite
**606**	GM_L	Composite

**607**	WM_L	Composite
**613**	GM_R	Composite
**614**	WM_R	Composite
**701**	TOTALBRAIN	Composite
**702**	ICV	Composite

Supplementary Table 5. Results from linear model used to examine regional volumetric differences between patients with first-episode depression (n=255) and healthy controls (n=558) with age, sex and years of education as covariates. Two-sided p values are presented along with FDR-corrected p values to account for multiple comparisons. Bold lettering indicates volume differences that are significant after FDR correction.

**ROI**	**t value**	**p value**	**FDR-corrected p value**
**Third Ventricle**	-1.07	0.29	0.40
**Fourth Ventricle**	-0.36	0.72	0.82
**Right Accumbens Area**	-0.75	0.45	0.57
**Left Accumbens Area**	-0.99	0.32	0.43
**Right Amygdala**	-1.87	0.06	0.12
**Left Amygdala**	0.19	0.85	0.90
**Brain Stem**	-0.53	0.60	0.71
**Right Caudate**	-0.57	0.57	0.68
**Left Caudate**	-0.80	0.42	0.53
**Right Cerebellum Exterior**	-2.15	0.03	0.07
**Left Cerebellum Exterior**	-2.36	0.02	0.05
**Right Cerebellum White Matter**	0.12	0.90	0.94
**Left Cerebellum White Matter**	0.05	0.96	0.97
**Right Hippocampus**	-0.29	0.77	0.85
**Left Hippocampus**	-0.86	0.39	0.51
**Right Inferior Lateral Ventricle**	-0.08	0.94	0.96
**Left Inferior Lateral Ventricle**	-0.04	0.97	0.98
**Right Lateral Ventricle**	-1.29	0.20	0.28
**Left Lateral Ventricle**	-0.66	0.51	0.63
**Right Pallidum**	-0.17	0.87	0.92
**Left Pallidum**	-0.01	0.99	0.99
**Right Putamen**	0.69	0.49	0.61
**Left Putamen**	0.29	0.77	0.85
**Right Thalamus Proper**	0.84	0.40	0.51
**Left Thalamus Proper**	-0.26	0.79	0.86
**Right Ventral DC**	-2.79	0.01	**0.02**
**Left Ventral DC**	-2.57	0.01	**0.03**
**Cerebellar Vermal Lobules I-V**	-3.17	0.002	**0.01**
**Cerebellar Vermal Lobules VI-VII**	0.98	0.33	0.44
**Cerebellar Vermal Lobules VIII-X**	-1.69	0.09	0.15
**Left Basal Forebrain**	-5.90	5.93E-9	**2.15E-7**
**Right Basal Forebrain**	-6.22	8.96E-10	**6.50E-8**
**Right frontal lobe WM**	-3.03	0.003	**0.01**
**Left frontal lobe WM**	-2.59	0.01	**0.03**

**Right occipital lobe WM**	-4.12	0.00004	**0.0004**
**Left occipital lobe WM**	-2.74	0.01	**0.02**
**Right parietal lobe WM**	-3.57	0.0004	**0.003**
**Left parietal lobe WM**	-3.83	0.0001	**0.001**
**Right temporal lobe WM**	-2.00	0.05	0.09
**Left temporal lobe WM**	-2.22	0.03	0.06
**Right fornix**	-0.30	0.76	0.85
**Left fornix**	-1.70	0.09	0.15
**Right anterior limb of internal capsule**	-0.39	0.70	0.80
**Left anterior limb of internal capsule**	0.89	0.37	0.49
**Right posterior limb of internal capsule inc. cerebral peduncle**	-3.02	0.003	**0.01**
**Left posterior limb of internal capsule inc. cerebral peduncle**	-2.66	0.01	**0.03**
**Corpus callosum**	-1.55	0.12	0.19
**Right anterior cingulate gyrus**	-4.55	6.38E-6	**0.0001**
**Left anterior cingulate gyrus**	-2.88	0.004	**0.02**
**Right anterior insula**	-2.35	0.02	0.05
**Left anterior insula**	-2.46	0.01	**0.04**
**Right anterior orbital gyrus**	-3.19	0.001	**0.01**
**Left anterior orbital gyrus**	-1.02	0.31	0.42
**Right angular gyrus**	-4.44	0.00001	**0.0001**
**Left angular gyrus**	-1.84	0.07	0.12
**Right calcarine cortex**	0.05	0.96	0.97
**Left calcarine cortex**	2.26	0.02	0.06
**Right central operculum**	-5.81	9.94E-9	**2.88E-7**
**Left central operculum**	-3.27	0.001	**0.01**
**Right cuneus**	-1.19	0.23	0.33
**Left cuneus**	-2.32	0.02	0.05
**Right entorhinal area**	-1.71	0.09	0.15
**Left entorhinal area**	-1.66	0.10	0.16
**Right frontal operculum**	-1.33	0.19	0.27
**Left frontal operculum**	-3.07	0.002	**0.01**
**Right frontal pole**	-4.59	5.23E-6	**0.0001**
**Left frontal pole**	-6.08	2.11E-9	**1.02E-7**
**Right fusiform gyrus**	-1.55	0.12	0.19
**Left fusiform gyrus**	-2.00	0.05	0.09
**Right gyrus rectus**	-4.99	7.74E-7	**0.00002**
**Left gyrus rectus**	-4.11	0.00004	**0.0004**
**Right inferior occipital gyrus**	1.83	0.07	0.12
**Left inferior occipital gyrus**	2.23	0.03	0.06
**Right inferior temporal gyrus**	-4.11	0.00005	**0.0004**

**Left inferior temporal gyrus**	-3.03	0.003	**0.01**
**Right lingual gyrus**	-2.41	0.02	**0.04**
**Left lingual gyrus**	-3.18	0.002	**0.01**
**Right lateral orbital gyrus**	-0.32	0.75	0.84
**Left lateral orbital gyrus**	-3.20	0.001	**0.01**
**Right middle cingulate gyrus**	-1.75	0.08	0.14
**Left middle cingulate gyrus**	-1.63	0.10	0.17
**Right medial frontal cortex**	-4.45	9.96E-6	**0.0001**
**Left medial frontal cortex**	-2.28	0.02	0.05
**Right middle frontal gyrus**	-3.13	0.002	**0.01**
**Left middle frontal gyrus**	-3.33	0.001	**0.01**
**Right middle occipital gyrus**	-2.61	0.01	**0.03**
**Left middle occipital gyrus**	-1.92	0.06	0.11
**Right medial orbital gyrus**	-3.38	0.001	**0.01**
**Left medial orbital gyrus**	0.20	0.84	0.90
**Right postcentral gyrus medial segment**	-1.77	0.08	0.14
**Left postcentral gyrus medial segment**	-4.13	0.00004	**0.0004**
**Right precentral gyrus medial segment**	-0.51	0.61	0.72
**Left precentral gyrus medial segment**	1.13	0.26	0.37
**Right superior frontal gyrus medial segment**	-1.02	0.31	0.42
**Left superior frontal gyrus medial segment**	-2.66	0.01	0.03
**Right middle temporal gyrus**	-2.29	0.02	0.05
**Left middle temporal gyrus**	-2.28	0.02	0.05
**Right occipital pole**	-2.36	0.02	0.05
**Left occipital pole**	-1.42	0.16	0.24
**Right occipital fusiform gyrus**	-0.65	0.52	0.63
**Left occipital fusiform gyrus**	-2.04	0.04	0.08
**Right opercular part of the inferior frontal gyrus**	-1.47	0.14	0.22
**Left opercular part of the inferior frontal gyrus**	-2.15	0.03	0.07
**Right orbital part of the inferior frontal gyrus**	-0.25	0.80	0.87
**Left orbital part of the inferior frontal gyrus**	0.86	0.39	0.51
**Right posterior cingulate gyrus**	-1.90	0.06	0.11
**Left posterior cingulate gyrus**	-0.63	0.53	0.64
**Right precuneus**	-1.41	0.16	0.24
**Left precuneus**	-3.10	0.002	**0.01**
**Right parahippocampal gyrus**	-1.70	0.09	0.15
**Left parahippocampal gyrus**	-1.67	0.10	0.16
**Right posterior insula**	-2.50	0.01	**0.04**
**Left posterior insula**	-3.66	0.0003	**0.002**
**Right parietal operculum**	2.34	0.02	0.05
**Left parietal operculum**	-1.31	0.19	0.27
**Right postcentral gyrus**	-3.31	0.001	**0.01**

**Left postcentral gyrus**	-1.94	0.05	0.10
**Right posterior orbital gyrus**	-1.57	0.12	0.19
**Left posterior orbital gyrus**	-3.12	0.002	**0.01**
**Right planum polare**	-2.95	0.003	**0.01**
**Left planum polare**	-3.43	0.001	**0.005**
**Right precentral gyrus**	-2.22	0.03	0.06
**Left precentral gyrus**	-2.21	0.03	0.06
**Right planum temporale**	0.42	0.67	0.77
**Left planum temporale**	-2.72	0.01	**0.02**
**Right subcallosal area**	1.43	0.15	0.23
**Left subcallosal area**	2.39	0.02	0.05
**Right superior frontal gyrus**	-2.43	0.02	**0.04**
**Left superior frontal gyrus**	-1.76	0.08	0.14
**Right supplementary motor cortex**	-0.14	0.89	0.93
**Left supplementary motor cortex**	-1.06	0.29	0.40
**Right supramarginal gyrus**	-0.48	0.63	0.73
**Left supramarginal gyrus**	-1.35	0.18	0.26
**Right superior occipital gyrus**	2.51	0.01	**0.04**
**Left superior occipital gyrus**	1.83	0.07	0.12
**Right superior parietal lobule**	-0.10	0.92	0.95
**Left superior parietal lobule**	-2.26	0.02	0.06
**Right superior temporal gyrus**	-2.41	0.02	**0.04**
**Left superior temporal gyrus**	-2.49	0.01	**0.04**
**Right temporal pole**	-6.57	1.01E-10	**1.47E-8**
**Left temporal pole**	-5.44	7.64E-8	**0.000002**
**Right triangular part of the inferior frontal gyrus**	-2.59	0.01	**0.03**
**Left triangular part of the inferior frontal gyrus**	-3.18	0.002	**0.01**
**Right transverse temporal gyrus**	-0.82	0.41	0.53
**Left transverse temporal gyrus**	-0.49	0.62	0.73

Supplementary References

Arnone, D., McKie, S., Elliott, R., Juhasz, G., Thomas, E. J., Downey, D., . . . Anderson, I. M. (2013). State-dependent changes in hippocampal grey matter in depression. *Mol Psychiatry, 18*(12), 1265-1272. doi:10.1038/mp.2012.150

Arnone, D., McKie, S., Elliott, R., Thomas, E. J., Downey, D., Juhasz, G., . . . Anderson, I. M. (2012). Increased amygdala responses to sad but not fearful faces in major depression: relation to mood state and pharmacological treatment. *Am J Psychiatry, 169*(8), 841-850. doi:10.1176/appi.ajp.2012.11121774

Dunlop, B. W., Binder, E. B., Cubells, J. F., Goodman, M. M., Kelley, M. E., Kinkead, B., . . . Mayberg, H. S. (2012). Predictors of remission in depression to individual and combined treatments (PReDICT): study protocol for a randomized controlled trial. *Trials, 13*, 106. doi:10.1186/1745-6215-13-106

Dutta, A., McKie, S., Downey, D., Thomas, E., Juhasz, G., Arnone, D., . . . Anderson, I. M. (2019). Regional default mode network connectivity in major depressive disorder: modulation by acute intravenous citalopram. *Transl Psychiatry, 9*(1), 116. doi:10.1038/s41398-019-0447-0

Ford, B. N., Yolken, R. H., Aupperle, R. L., Teague, T. K., Irwin, M. R., Paulus, M. P., & Savitz, J. (2019). Association of Early-Life Stress With Cytomegalovirus Infection in Adults With Major Depressive Disorder. *JAMA Psychiatry, 76*(5), 545-547. doi:10.1001/jamapsychiatry.2018.4543

Godlewska, B. R., Browning, M., Norbury, R., Igoumenou, A., Cowen, P. J., & Harmer, C. J. (2018). Predicting Treatment Response in Depression: The Role of Anterior Cingulate Cortex. *International Journal of Neuropsychopharmacology, 21*(11), 988-996. doi:10.1093/ijnp/pyy069

Godlewska, B. R., Hasselmann, H. W., Igoumenou, A., Norbury, R., & Cowen, P. J. (2014). Short-term escitalopram treatment and hippocampal volume. *Psychopharmacology (Berl), 231*(23), 4579-4581. doi:10.1007/s00213-014-3771-3

Green, S., Lambon Ralph, M. A., Moll, J., Deakin, J. F., & Zahn, R. (2012). Guilt-selective functional disconnection of anterior temporal and subgenual cortices in major depressive disorder. *Arch Gen Psychiatry, 69*(10), 1014-1021. doi:10.1001/archgenpsychiatry.2012.135

Habota, T., Sandu, A. L., Waiter, G. D., McNeil, C. J., Steele, J. D., Macfarlane, J. A., . . . McIntosh, A. M. (2019). Cohort profile for the STratifying Resilience and Depression Longitudinally (STRADL) study: A depression-focused investigation of Generation Scotland, using detailed clinical, cognitive, and neuroimaging assessments. *Wellcome Open Res, 4*, 185. doi:10.12688/wellcomeopenres.15538.2

Hamilton, M. (1960). A rating scale for depression. *J Neurol Neurosurg Psychiatry, 23*(1), 56-62. doi:10.1136/jnnp.23.1.56

Leucht, S., Fennema, H., Engel, R. R., Kaspers-Janssen, M., & Szegedi, A. (2018). Translating the HAM-D into the MADRS and vice versa with equipercentile linking. *J Affect Disord, 226*, 326-331. doi:10.1016/j.jad.2017.09.042

MacQueen, G. M., Hassel, S., Arnott, S. R., Jean, A., Bowie, C. R., Bray, S. L., . . . Kennedy, S. H. (2019). The Canadian Biomarker Integration Network in Depression (CAN-BIND): magnetic resonance imaging protocols. *J Psychiatry Neurosci, 44*(4), 223-236. doi:10.1503/jpn.180036

Misaki, M., Suzuki, H., Savitz, J., Drevets, W. C., & Bodurka, J. (2016). Individual Variations in Nucleus Accumbens Responses Associated with Major Depressive Disorder Symptoms. *Sci Rep, 6*, 21227. doi:10.1038/srep21227

Montgomery, S. A., & Asberg, M. (1979). A new depression scale designed to be sensitive to change. *Br J Psychiatry, 134*, 382-389. doi:10.1192/bjp.134.4.382

Nouretdinov, I., Costafreda, S. G., Gammerman, A., Chervonenkis, A., Vovk, V., Vapnik, V., & Fu, C. H. (2011). Machine learning classification with confidence: application of transductive conformal predictors to MRI-based diagnostic and prognostic markers in depression. *Neuroimage, 56*(2), 809-813. doi:10.1016/j.neuroimage.2010.05.023

Qiu, L., Xia, M., Cheng, B., Yuan, L., Kuang, W., Bi, F., . . . Gong, Q. (2018). Abnormal dynamic functional connectivity of amygdalar subregions in untreated patients with first-episode major depressive disorder. *J Psychiatry Neurosci, 43*(4), 262-272. doi:10.1503/jpn.170112

Rush, A. J., Trivedi, M. H., Ibrahim, H. M., Carmody, T. J., Arnow, B., Klein, D. N., . . . Keller, M. B. (2003). The 16-Item Quick Inventory of Depressive Symptomatology (QIDS), clinician rating (QIDS-C), and self-report (QIDS-SR): a psychometric evaluation in patients with chronic major depression. *Biol Psychiatry, 54*(5), 573-583. doi:10.1016/s0006-3223(02)01866-8

Sacchet, M. D., & Gotlib, I. H. (2017). Myelination of the brain in Major Depressive Disorder: An in vivo quantitative magnetic resonance imaging study. *Sci Rep, 7*(1), 2200. doi:10.1038/s41598-017-02062-y

Sacchet, M. D., Livermore, E. E., Iglesias, J. E., Glover, G. H., & Gotlib, I. H. (2015). Subcortical volumes differentiate Major Depressive Disorder, Bipolar Disorder, and remitted Major Depressive Disorder. *Journal of Psychiatric Research, 68*, 91-98. doi:https://doi.org/10.1016/j.jpsychires.2015.06.002

Sankar, A., Zhang, T., Gaonkar, B., Doshi, J., Erus, G., Costafreda, S. G., . . . Fu, C. H. (2016). Diagnostic potential of structural neuroimaging for depression from a multi-ethnic community sample. *BJPsych Open, 2*(4), 247-254. doi:10.1192/bjpo.bp.115.002493

Schwartz, J., Ordaz, S. J., Kircanski, K., Ho, T. C., Davis, E. G., Camacho, M. C., & Gotlib, I. H. (2019). Resting-state functional connectivity and inflexibility of daily emotions in major depression. *J Affect Disord, 249*, 26-34. doi:10.1016/j.jad.2019.01.040

Trivedi, M. H., McGrath, P. J., Fava, M., Parsey, R. V., Kurian, B. T., Phillips, M. L., . . . Weissman, M. M. (2016). Establishing moderators and biosignatures of antidepressant response in clinical care (EMBARC): Rationale and design. *Journal of Psychiatric Research, 78*, 11-23. doi:https://doi.org/10.1016/j.jpsychires.2016.03.001

Trivedi, M. H., Rush, A. J., Ibrahim, H. M., Carmody, T. J., Biggs, M. M., Suppes, T., . . . Kashner, T. M. (2004). The Inventory of Depressive Symptomatology, Clinician Rating (IDS-C) and Self-Report (IDS-SR), and the Quick Inventory of Depressive Symptomatology, Clinician Rating (QIDS-C) and Self-Report (QIDS-SR) in public sector patients with mood disorders: a psychometric evaluation. *Psychol Med, 34*(1), 73-82. doi:10.1017/s0033291703001107

Uher, R., Farmer, A., Maier, W., Rietschel, M., Hauser, J., Marusic, A., . . . Aitchison, K. J. (2008). Measuring depression: comparison and integration of three scales in the GENDEP study. *Psychol Med, 38*(2), 289-300. doi:10.1017/s0033291707001730

Wise, T., Marwood, L., Perkins, A. M., Herane-Vives, A., Joules, R., Lythgoe, D. J., . . . Arnone, D. (2017). Instability of default mode network connectivity in major depression: a two-sample confirmation study. *Transl Psychiatry, 7*(4), e1105. doi:10.1038/tp.2017.40

Wise, T., Marwood, L., Perkins, A. M., Herane-Vives, A., Williams, S. C. R., Young, A. H., . . . Arnone, D. (2018). A morphometric signature of depressive symptoms in unmedicated patients with mood disorders. *Acta Psychiatr Scand, 138*(1), 73-82. doi:10.1111/acps.12887

Zhao, Y., Niu, R., Lei, D., Shah, C., Xiao, Y., Zhang, W., . . . Gong, Q. (2020). Aberrant Gray Matter Networks in Non-comorbid Medication-Naive Patients With Major Depressive Disorder and Those With Social Anxiety Disorder. *Front Hum Neurosci, 14*, 172. doi:10.3389/fnhum.2020.00172

Zhao, Y., Zhang, F., Zhang, W., Chen, L., Chen, Z., Lui, S., & Gong, Q. (2021). Decoupling of Gray and White Matter Functional Networks in Medication-Naïve Patients With Major Depressive Disorder. *J Magn Reson Imaging, 53*(3), 742-752. doi:10.1002/jmri.27392

Zheng, H., Ford, B. N., Bergamino, M., Kuplicki, R., Hunt, P. W., Bodurka, J., . . . Savitz, J. (2021). A hidden menace? Cytomegalovirus infection is associated with reduced cortical gray matter volume in major depressive disorder. *Mol Psychiatry, 26*(8), 4234-4244. doi:10.1038/s41380-020-00932-y
