# Supplementary material for: Neuroanatomical dimensions in medication-free individuals with major depressive disorder and treatment response to SSRI antidepressant medications or placebo
Source: Nat Ment Health. 2024 Jan 12;2(2):164–76. doi: 10.1038/s44220-023-00187-w (PMC11211072; doi:10.1038/s44220-023-00187-w)
Supplement: Supplementary file 2 — Reporting Summary [file 44220_2023_187_MOESM2_ESM.pdf]

nature portfolio

Corresponding author(s): Mathilde Antoniades

Last updated by author(s): Feb 15, 2023

Reporting Summary

Nature Portfolio wishes to improve the reproducibility of the work that we publish. This form provides structure for consistency and transparency in reporting. For further information on Nature Portfolio policies, see our Editorial Policies and the Editorial Policy Checklist.

Statistics

For all statistical analyses, confirm that the following items are present in the figure legend, table legend, main text, or Methods section.

n/a	Confirmed	
☐	☒	The exact sample size (*n*) for each experimental group/condition, given as a discrete number and unit of measurement
☒	☐	A statement on whether measurements were taken from distinct samples or whether the same sample was measured repeatedly
☐	☒	The statistical test(s) used AND whether they are one- or two-sided<br>*Only common tests should be described solely by name; describe more complex techniques in the Methods section.*
☐	☒	A description of all covariates tested
☒	☐	A description of any assumptions or corrections, such as tests of normality and adjustment for multiple comparisons
☐	☒	A full description of the statistical parameters including central tendency (e.g. means) or other basic estimates (e.g. regression coefficient) AND variation (e.g. standard deviation) or associated estimates of uncertainty (e.g. confidence intervals)
☐	☒	For null hypothesis testing, the test statistic (e.g. *F*, *t*, *r*) with confidence intervals, effect sizes, degrees of freedom and *P* value noted<br>*Give P values as exact values whenever suitable.*
☒	☐	For Bayesian analysis, information on the choice of priors and Markov chain Monte Carlo settings
☒	☐	For hierarchical and complex designs, identification of the appropriate level for tests and full reporting of outcomes
☐	☒	Estimates of effect sizes (e.g. Cohen's *d*, Pearson's *r*), indicating how they were calculated

Our web collection on statistics for biologists contains articles on many of the points above.

Software and code

Policy information about availability of computer code

Data collection	No software was used for data collection
Data analysis	Python version 3.9.7: statsmodels version 0.13.1<br>R version 4.2.2: WebPower 0.8.6; effectsize 0.8.2; ggplot2 3.4.0<br>MATLAB 2018A<br>MUSE for image segmentation available at: https://www.nitrc.org/projects/cbica_muse<br>HYDRA software available at: https://github.com/evarol/HYDRA

For manuscripts utilizing custom algorithms or software that are central to the research but not yet described in published literature, software must be made available to editors and reviewers. We strongly encourage code deposition in a community repository (e.g. GitHub). See the Nature Portfolio guidelines for submitting code & software for further information.

Data

Policy information about availability of data

All manuscripts must include a data availability statement. This statement should provide the following information, where applicable:

- Accession codes, unique identifiers, or web links for publicly available datasets
- A description of any restrictions on data availability
- For clinical datasets or third party data, please ensure that the statement adheres to our policy

Derived brain measures can be accessed on reasonable request to C.Fu@uel.ac.uk and Christos.davatzikos@Pennmedicine.upenn.edu.

Human research participants

Policy information about studies involving human research participants and Sex and Gender in Research.

Reporting on sex and gender	In our study we report data on sex, not gender as the latter was not collected.<br>We include 404 female and 295 male healthy controls. There are 439 females and 246 male MDD subjects.<br>Sex was included as a covariate in the linear models as there were significantly more females than males in our sample.
Population characteristics	The population consists of healthy controls and patients with major depressive disorder. Our sample has significantly more females than males and the age range is between 16-72 for controls and 18-65 for patients. All patients were medication-free at the time of scanning. The number of MDD participants who were treatment-naïve is 128. A subset were part of clinical trials and took one of the following selective serotonin reuptake inhibitor antidepressant medications: sertraline, escitalopram, citalpram or placebo.
Recruitment	Data was already collected - our study includes consortium data
Ethics oversight	Each study was approved by the local ethics committe

Note that full information on the approval of the study protocol must also be provided in the manuscript.

Field-specific reporting

Please select the one below that is the best fit for your research. If you are not sure, read the appropriate sections before making your selection.

☐ Life sciences ☒ Behavioural & social sciences ☐ Ecological, evolutionary & environmental sciences

For a reference copy of the document with all sections, see nature.com/documents/nr-reporting-summary-flat.pdf

Behavioural & social sciences study design

All studies must disclose on these points even when the disclosure is negative.

Study description	Case-control
Research sample	The present study consists of a total of 685 MDD participants from 10 studies: Canadian Biomarker Integration Network in Depression (CAN-BIND) (N=92, (MacQueen et al., 2019)), Establishing Moderators and Biosignatures of Antidepressant Response in Clinical Care (EMBARC) (N=257, (Madhukar H. Trivedi et al., 2016)), Huaxi MR Research Center SCU (HMRRC) (N=111, (J. Zhang et al., 2011)), King's College London (KCL) (N=20, (Wise et al., 2018)), Manchester Remedi (N=40, (Arnone, McIntosh, Ebmeier, Munafò, & Anderson, 2012; Dutta et al., 2019)), Laureate Institute for Brain Research (LIBR) (N =554, (Misaki, Suzuki, Savitz, Drevets, & Bodurka, 2016; Victor et al., 2018)), Oxford (N=39, (Vai et al., 2016)), Predictors of Remission in Depression to Individual and Combined Treatments (PReDICT) (N=63, (Dunlop et al., 2012)), Stanford SNAP (N=8, (Sacchet, Livermore, Iglesias, Glover, & Gotlib, 2015)), STratifying Resilience and Depression Longitudinally (STRADL) (N=1, (Habota et al., 2019)); and a total of 699 healthy control (HC) participants from 10 studies: CAN-BIND (N=23), EMBARC (N=39), KCL (N=20), LIBR (N=141), Manchester Blame (N=46), Manchester Remedi (N=30), Oxford (N=31), HMRRC SCU (N=139), Stanford SNAP (N=50), STRADL (N=180).<br><br>The rationale and design of the consortium is described here:<br>Fu CHY, Erus G, Fan Y, et al. AI-based dimensional neuroimaging system for characterizing heterogeneity in brain structure and function in major depressive disorder: COORDINATE-MDD consortium design and rationale. BMC Psychiatry. Jan 23 2023;23(1):59. doi:10.1186/s12888-022-04509-7
Sampling strategy	Of the larger consortium dataset, we included individuals for this analysis based on the following criteria:<br>1) DSM-based diagnosis of MDD<br>2) in current depressive episode of at least moderate severity, defined as a 17-item Hamilton Rating Scale for Depression score equal to or greater than 14<br>3) medication-free at the time of scanning

	4) no current comorbid psychiatric, medical or neurological disorders<br>5) no treatment resistant depression, defined as not achieving clinical response to two or more trials of antidepressant medications.
Data collection	Data was not collected in this study- our study includes consortium data<br>Researchers are aware of which subjects are patients and which are healthy
Timing	Data was not collected in this study- our study includes consortium data
Data exclusions	See inclusion and exclusion criteria in the 'Sampling Strategy' section
Non-participation	Data was not collected in this study- our study includes consortium data
Randomization	Participants were not allocated into experimental groups for this study- we included healthy subjects (no DSM diagnoses) and patients diagnosed with major depressive disorder by trained clinicians.

Reporting for specific materials, systems and methods

We require information from authors about some types of materials, experimental systems and methods used in many studies. Here, indicate whether each material, system or method listed is relevant to your study. If you are not sure if a list item applies to your research, read the appropriate section before selecting a response.

Materials & experimental systems

n/a	Involved in the study
☒	☐ Antibodies
☒	☐ Eukaryotic cell lines
☒	☐ Palaeontology and archaeology
☒	☐ Animals and other organisms
☒	☐ Clinical data
☒	☐ Dual use research of concern

Methods

n/a	Involved in the study
☒	☐ ChIP-seq
☒	☐ Flow cytometry
☐	☒ MRI-based neuroimaging

Magnetic resonance imaging

Experimental design

Design type	Consortium data pooling, data from multiple sources have been combined into a single dataset
Design specifications	TMRI data from participants with the following characteristics:<br>1) DSM-based diagnosis of MDD<br>2) In current depressive episode of at least moderate severity, defined as a 17-item Hamilton Rating Scale for Depression score equal to or greater than 14<br>3) Medication-free at the time of scanning<br>4) No current comorbid psychiatric, medical or neurological disorders<br>5) Not having treatment resistant depression, defined as not achieving clinical response to two or more trials of antidepressant medications.<br>This study pooled together data that had already been acquired from the original study sites.
Behavioral performance measures	MRI data was pooled from original studies in which various behavioral performance measures had been acquired.

Acquisition

Imaging type(s)	Structural
Field strength	3T and 1.5T
Sequence & imaging parameters	The parameters for each study are described in the supplementary material
Area of acquisition	Whole brain scan

Diffusion MRI ☐ Used ☒ Not used

Preprocessing

Preprocessing software	Brain mask, segmentation and ROI volumes calculated using MUSE, regional volumetric analysis using RAVENS (Davatzikos et al., 2001)<br>HYDRA (https://github.com/evarol/HYDRA) was run using MATLAB 2018A<br>MUSE software available at: https://www.nitrc.org/projects/cbica_muse

Normalization	Non-linear transformation
Normalization template	Registration to common space using multiple atlases during MUSE segmentation
Noise and artifact removal	N4 Bias Field Correction, FAST Bias Field Correction
Volume censoring	None

Statistical modeling & inference

Model type and settings	MIDAS - Multivariate voxel-wise analysis of group differences using locally linear discriminative learning
Effect(s) tested	MIDAS used to identify significance and size of group differences in estimated brain volumes between each subtype and controls

Specify type of analysis: ☒ Whole brain ☐ ROI-based ☐ Both

Statistic type for inference (See Eklund et al. 2016)	Voxel-wise
Correction	FDR

Models & analysis

n/a	Involved in the study
☒	☐ Functional and/or effective connectivity
☒	☐ Graph analysis
☐	☒ Multivariate modeling or predictive analysis

Multivariate modeling and predictive analysis	HYDRA used for identifying disease subtypes from extracted ROI volumes, age, sex, and diagnosis data. Cluster stability (the Adjusted Rand Index) was used to evaluate model performance and optimal number of subtypes.
